# Genome-wide association study identifies multiple susceptibility loci for multiple myeloma

**DOI:** 10.1038/ncomms12050

**Published:** 2016-07-01

**Authors:** Jonathan S. Mitchell, Ni Li, Niels Weinhold, Asta Försti, Mina Ali, Mark van Duin, Gudmar Thorleifsson, David C. Johnson, Bowang Chen, Britt-Marie Halvarsson, Daniel F. Gudbjartsson, Rowan Kuiper, Owen W. Stephens, Uta Bertsch, Peter Broderick, Chiara Campo, Hermann Einsele, Walter A. Gregory, Urban Gullberg, Marc Henrion, Jens Hillengass, Per Hoffmann, Graham H. Jackson, Ellinor Johnsson, Magnus Jöud, Sigurður Y. Kristinsson, Stig Lenhoff, Oleg Lenive, Ulf-Henrik Mellqvist, Gabriele Migliorini, Hareth Nahi, Sven Nelander, Jolanta Nickel, Markus M. Nöthen, Thorunn Rafnar, Fiona M. Ross, Miguel Inacio da Silva Filho, Bhairavi Swaminathan, Hauke Thomsen, Ingemar Turesson, Annette Vangsted, Ulla Vogel, Anders Waage, Brian A. Walker, Anna-Karin Wihlborg, Annemiek Broyl, Faith E. Davies, Unnur Thorsteinsdottir, Christian Langer, Markus Hansson, Martin Kaiser, Pieter Sonneveld, Kari Stefansson, Gareth J. Morgan, Hartmut Goldschmidt, Kari Hemminki, Björn Nilsson, Richard S. Houlston

**Affiliations:** 1 Division of Genetics and Epidemiology, The Institute of Cancer Research, 15 Cotswold Road, Sutton, Surrey SM2 5NG, UK; 2 Myeloma Institute for Research and Therapy, University of Arkansas for Medical Sciences, Little Rock, Arkansas 72205, USA; 3 Department of Internal Medicine V, University of Heidelberg, 69117 Heidelberg, Germany; 4 German Cancer Research Center, 69120 Heidelberg, Germany; 5 Center for Primary Health Care Research, Lund University, SE-205 02 Malmo, Sweden; 6 Hematology and Transfusion Medicine, Department of Laboratory Medicine, BMC B13, SE-221 84 Lund, Sweden; 7 Department of Hematology, Erasmus MC Cancer Institute, 3075 EA Rotterdam, The Netherlands; 8 deCODE Genetics, Sturlugata 8, IS-101 Reykjavik, Iceland; 9 Division of Molecular Pathology, The Institute of Cancer Research, Surrey SM2 5NG, UK; 10 School of Engineering and Natural Sciences, University of Iceland, IS-101 Reykjavik, Iceland; 11 National Centre of Tumor Diseases, 69120 Heidelberg, Germany; 12 University Clinic of Würzburg, 97080 Würzburg, Germany; 13 Clinical Trials Research Unit, University of Leeds, Leeds LS2 9PH, UK; 14 Institute of Human Genetics, University of Bonn, D-53127 Bonn, Germany; 15 Division of Medical Genetics, Department of Biomedicine, University of Basel, 4003 Basel, Switzerland; 16 Royal Victoria Infirmary, Newcastle upon Tyne, NE1 4LP, UK; 17 Clinical Immunology and Transfusion Medicine, Laboratory Medicine, Office of Medical Services, SE-221 85 Lund, Sweden; 18 Department of Hematology, Landspitali, National University Hospital of Iceland, IS-101 Reykjavik, Iceland; 19 Hematology Clinic, Skåne University Hospital, SE-221 85 Lund, Sweden; 20 Section of Hematology, Sahlgrenska University Hospital, Gothenburg 413 45, Sweden; 21 Center for Hematology and Regenerative Medicine, Karolinska Institutet, SE-171 77 Stockholm, Sweden; 22 Rudbeck Laboratory, Department of Immunology, Pathology and Genetics, Uppsala University, SE-751 05 Uppsala, Sweden; 23 Department of Genomics, Life & Brain Center, University of Bonn, D-53127 Bonn, Germany; 24 Wessex Regional Genetics Laboratory, University of Southampton, Salisbury SP2 8BJ, UK; 25 Department of Haematology, University Hospital of Copenhagen at Rigshospitalet, Blegdamsvej 9, DK-2100 Copenhagen, Denmark; 26 National Research Centre for the Working Environment, DK-2100 Copenhagen, Denmark; 27 Department of Cancer Research and Molecular Medicine, Norwegian University of Science and Technology, Box 8905, N-7491 Trondheim, Norway; 28 Faculty of Medicine, University of Iceland, IS-101 Reykjavik, Iceland; 29 Department of Internal Medicine III, University of Ulm, D-89081 Ulm, Germany; 30 Broad Institute, 7 Cambridge Center, Cambridge, Massachusetts 02142, USA

## Abstract

Multiple myeloma (MM) is a plasma cell malignancy with a significant heritable basis. Genome-wide association studies have transformed our understanding of MM predisposition, but individual studies have had limited power to discover risk loci. Here we perform a meta-analysis of these GWAS, add a new GWAS and perform replication analyses resulting in 9,866 cases and 239,188 controls. We confirm all nine known risk loci and discover eight new loci at 6p22.3 (rs34229995, *P*=1.31 × 10^−8^), 6q21 (rs9372120, *P*=9.09 × 10^−15^), 7q36.1 (rs7781265, *P*=9.71 × 10^−9^), 8q24.21 (rs1948915, *P*=4.20 × 10^−11^), 9p21.3 (rs2811710, *P*=1.72 × 10^−13^), 10p12.1 (rs2790457, *P*=1.77 × 10^−8^), 16q23.1 (rs7193541, *P*=5.00 × 10^−12^) and 20q13.13 (rs6066835, *P*=1.36 × 10^−13^), which localize in or near to *JARID2*, *ATG5*, *SMARCD3*, *CCAT1*, *CDKN2A*, *WAC*, *RFWD3* and *PREX1*. These findings provide additional support for a polygenic model of MM and insight into the biological basis of tumour development.

## Abstract (web summary)

Previous genome-wide association studies have identified loci associated with the risk of multiple myeloma. Here, the authors present a meta-analysis of six genome wide association studies of the disease and identify eight new loci; functional studies identify genes as candidates for the basis of these associations.

## Graphical abstract

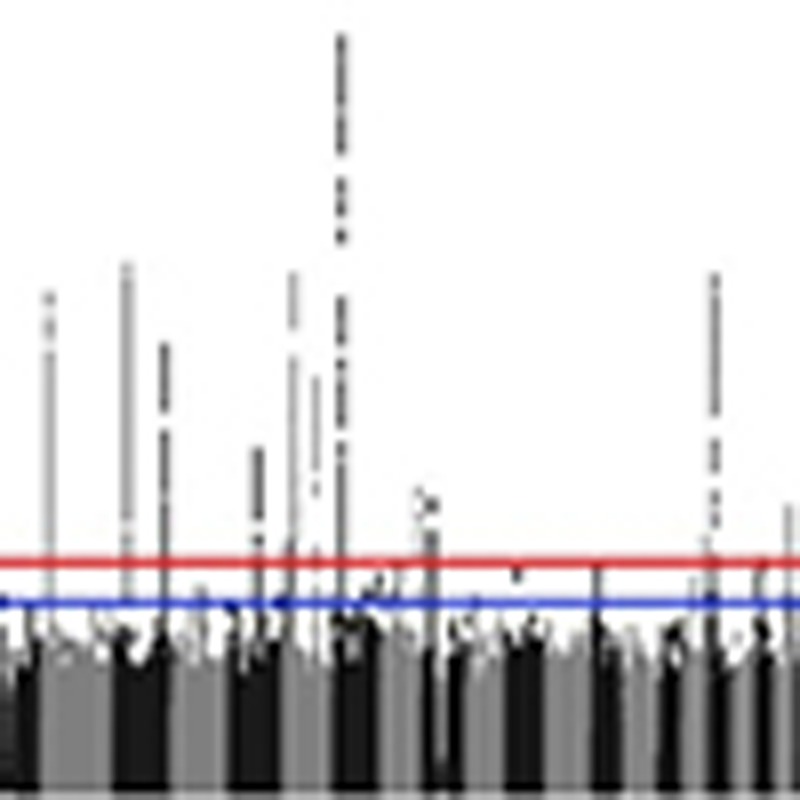

Multiple myeloma (MM) is a malignancy of plasma cells that has a significant genetic component as evidenced by the two- to fourfold increased risk shown in relatives of MM patients^1^. Our understanding of MM susceptibility has been transformed by recent genome-wide association studies (GWASs), which have identified the first risk alleles for MM^2^,^3^,^4^,^5^ and its precursor condition monoclonal gammopathy of unknown significance^5^. Although projections indicate that additional risk variants for MM can be discovered by GWAS^6^, the statistical power of these individual studies is limited.

To gain comprehensive insight into MM predisposition, we performed a meta-analysis of these GWAS, new GWAS and replication comprising 9,866 cases and 239,188 controls. We confirmed all nine known risk loci and discovered eight new risk loci for MM. Our findings provide further insights into the genetic and biological basis of MM predisposition.

## Results

### Association analysis

**Figure 1:**
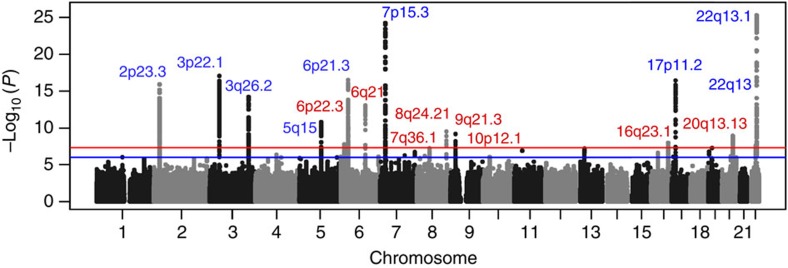
Manhattan plot of association *P*-values. Shown are the genome-wide *P*-values (two sided) of 12.4 million successfully imputed autosomal SNPs in 7,319 cases and 234,385 controls from the discovery phase. Labelled in blue are previously identified risk loci and labelled in red are newly identified risk loci. The red horizontal line represents the genome-wide significance threshold of *P*=5.0 × 10^−8^ and the blue horizontal line represents the threshold of *P*=1.0 × 10^−6^ used to define promising SNPs.

To identify new MM susceptibility loci, we analysed genome-wide association data from six populations of European ancestry (Supplementary Tables 1 and 2): a new sample set from the Netherlands, two previously reported sample sets from United Kingdom and Germany, to which we added additional cases^2^, and three previously published sample sets from Sweden/Norway, Iceland and the Unites States^5^,^7^. After filtering, the six studies provided single-nucleotide polymorphism (SNP) microarray genotypes on 7,319 cases and 234,385 controls (Supplementary Tables 1 and 2). To increase genomic resolution, we imputed >10 million SNPs using either the 1,000 Genomes Project^8^ combined with UK10K^9^ (MM data sets from the Netherlands, United Kingdom, Germany, Sweden/Norway and the United States) or deCODE Genetics (MM data set from Iceland^10^) as reference. Quantile–quantile plots for SNPs with minor allele frequency (MAF)>0.5% post imputation did not show evidence of substantive overdispersion (*λ*=1.00–1.06; Supplementary Fig. 1). Pooling association testing results from the six sample sets, we derived joint odds ratios and 95% confidence intervals under a fixed-effects model for each SNP and associated per allele *P*-value. In this analysis, associations for all nine established risk loci showed a consistent direction of effect with previously reported studies and have *P*<5.0 × 10^−8^ (Fig. 1 and Supplementary Table 3).

We identified 315 SNPs at 16 loci that showed evidence of association (*P*<1.0 × 10^−6^) not previously implicated in the risk of developing MM (Fig. 1 and Supplementary Tables 4 and 5). For 13 of the 16 loci, the strongest signal was provided by an imputed SNP. We confirmed the fidelity of imputation for 12 of the 13 imputed SNPs in multiple series (Supplementary Tables 6 and 7; rs78311596 imputation unconfirmed). Using allele-specific PCR, we genotyped the 15 substantiated SNPs in additional UK, Germany, Sweden/Norway and Denmark sample series totalling 2,547 cases and 4,803 controls. Meta-analysing the discovery and replication samples, we identified genome-wide significant associations for MM with eight previously unreported loci (Table 1 and Supplementary Tables 8 and 9) at 6p22.3 (rs34229995, *P*=1.31 × 10^−8^), 6q21 (rs9372120, *P*=9.09 × 10^−15^), 7q36.1 (rs7781265, *P*=9.71 × 10^−9^), 8q24.21 (rs1948915, *P*=4.20 × 10^−11^), 9p21.3 (rs2811710, *P*=1.72 × 10^−13^), 10p12.1 (rs2790457, *P*=1.77 × 10^−8^), 16q23.1 (rs7193541, *P*=5.00 × 10^−12^) and 20q13.13 (rs6066835, *P*=1.36 × 10^−13^). We also observed two promising associations (that is, *P*<5.0 × 10^−7^) at 6q27 (rs1034447) and at 7q22.3 (rs17507636) (Supplementary Tables 8 and 9). Conditional analysis of GWAS data showed no evidence for additional independent signals at the loci.

**Figure 2:**
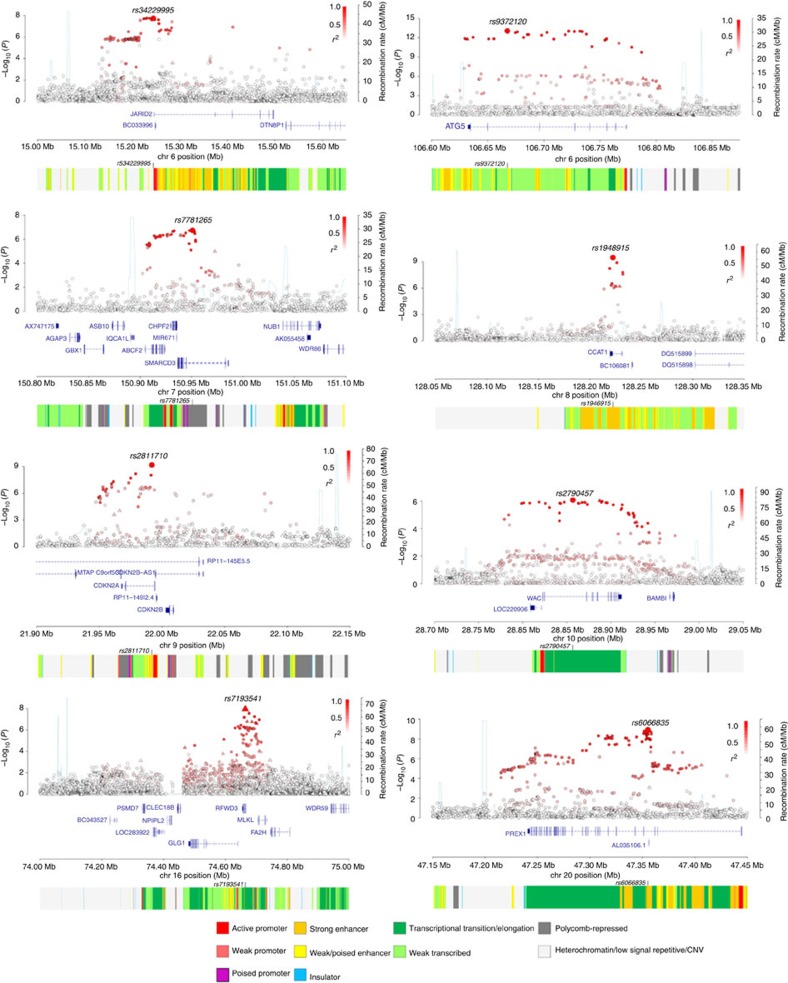
Regional plots of association results and recombination rates for the newly identified risk loci for multiple myeloma. Results for 6p22.3 (rs34229995), 6q21 (rs9372120), 7q36.1 (rs7781265), 8q24.21 (rs1948915), 9p21.3 (rs2811710), 10p12.1 (rs2790457), 16q23.1 (rs7193541) and 20q13.13 (rs6066835). Plots (using visPig^70^) show association results of both genotyped (triangles) and imputed (circles) SNPs in the GWAS samples and recombination rates. −Log_10_ *P*-values (*y* axes) of the SNPs are shown according to their chromosomal positions (*x* axes). The sentinel SNP in each combined analysis is shown as a large circle or triangle and is labelled by its rsID. The colour intensity of each symbol reflects the extent of LD with the top SNP, white (*r*^2^=0) through to dark red (*r*^2^=1.0). Genetic recombination rates, estimated using 1,000 Genomes Project samples, are shown with a light blue line. Physical positions are based on NCBI build 37 of the human genome. Also shown are the relative positions of genes and transcripts mapping to the region of association. Genes have been redrawn to show their relative positions; therefore, maps are not to physical scale. On the bottom is the chromatin-state segmentation track (ChromHMM) for lymphoblastoid cells using data from the HapMap ENCODE Project.

The 6q21 association marked by rs9372120 (Fig. 2) maps to intron 6 of *ATG5* (*Homo sapiens* autophagy related 5). The 8q24.21 variant rs1948915 maps to *CCAT1* (colon cancer-associated transcript 1; Fig. 2). The same region at 8q24.21 harbours multiple independent loci with different tumour specificities^11^, including the B-cell malignancies diffuse B-cell lymphoma^12^, Hodgkin's lymphoma^13^ and chronic lymphocytic leukaemia^14^. With the possible exception of chronic lymphocytic leukaemia, the linkage disequilibrium (LD) blocks defining these identified cancer risk loci are distinct from the 8q24.21 MM association signal (pairwise LD metrics *r*^2^<0.03; Supplementary Table 10). The 9p21.3 variant rs2811710 maps to intron 1 of *CDKN2A/p16INK4A* (cyclin-dependent kinase inhibitor 2A, Fig. 2). Although the 9p21.3 region is a susceptibility locus for multiple tumour types including breast and lung cancer, glioma and acute lymphoblastic leukaemia^15^, the rs2811710 association for MM is distinct (Supplementary Table 11). The 16q23.1 (rs7193541) association is a non-synonymous SNP I564V of *RFWD3* (encoding ring finger WD domain 3; Fig. 2). 6p22.3 (rs34229995) and 7q36.1 (rs7781265) associations mark chromatin-regulating genes; rs34229995 is 2.2-kb telomeric to the 5′ of *JARID2* (jumonji, AT-rich interactive domain 2; Fig. 2) and rs7781265 localizing to intron 2 of *SMARCD3* (swi/snf-related, matrix-associated, actin-dependent regulator of chromatin, subfamily d, member 3; Fig. 2). The 10p12.1 (rs2790457) association localizes to intron 3 of the gene encoding *WAC* (ww domain-containing adaptor with coiled-coil region), which has recently been shown to be part of an extended autophagy network^16^. The 20q13.13 (rs6066835) association mapped to intron 3 of *PREX1* (phosphatidylinositol-3, 4, 5-trisphosphate-dependent Rac exchange factor 1) (Fig. 2).

### Relationship between the new MM SNPs and phenotype

We tested for associations between sex or age at diagnosis and genotype for each of the eight risk SNPs by case-only analysis using all individuals in five of the six sample sets and observed no such relationships (Supplementary Tables 12 and 13). In addition, case-only analysis provided no evidence for associations between risk SNPs and cytogenetic MM subtype (Supplementary Table 14) or MM-specific overall survival (Supplementary Table 15). Collectively, these data are compatible with the risk variants having generic effects on MM development rather than tumour progression.

### Biological inference

To the extent that they have been deciphered, many of the GWAS loci map to non-coding regions of the genome and influence gene regulation. In this respect, it is perhaps not surprising that none of the genes annotated by the GWAS signals we identify are somatically mutated in MM (Supplementary Table 16). Hence, to gain insight into the biological mechanisms for the associations at the eight newly identified risk SNPs, we first performed expression quantitative trait loci (eQTL) analysis using gene expression profiles of CD138-positive MM plasma cells from the United Kingdom (*n*=183), Germany (*n*=658) and the United States (*n*=608) cases (Affymetrix Human Genome U133 2.0 Plus Array; NCBI GEO Data sets GSE21349, GSE31161, GSE2658 and EBI ArrayExpress E-MTAB-2299). In addition, we interrogated publicly accessible expression data on whole blood, adipocytes, skin cells and lymphoblastoid cell lines (LCLs). To explore methylation QTL (meQTLs) at each risk locus, we analysed Illumina Infinium HumanMethylation450 BeadChip data on CD138-positive MM plasma cells from 365 UK patients. In MM plasma cells, we identified significant associations between rs2790457 and decreased expression of *WAC* (*P*=6.58 × 10^−24^) and rs6066835, and increased expression of *PREX1* (*P*=3.85 × 10^−5^) (Supplementary Fig. 2 and Supplementary Data 1). We also detected strong *cis*-meQTLs at *WAC* and *PREX1* with rs2790457 and rs6066835 genotypes (*P*-values 1.42 × 10^−6^ and 1.12 × 10^−4^, respectively; Supplementary Data 1). The direction of these eQTLs and meQTLs is compatible with the 10p12.1 signal encompassing an active promotor for *WAC*, whereas the 20q13.13 signal does not capture an active promotor in the gene body of *PREX1* (Fig. 2).

DNA methylation plays a central role in epigenetic regulation of gene expression; however, meQTLs and *cis*-acting eQTLs do not always overlap. Thus, although rs7193541 showed a strong meQTL for *RFWD3* methylation and reduced expression of *RFWD3* in whole blood, no eQTL was shown in MM plasma cells (Supplementary Data 1).

Various lines of evidence indicate that chromatin lopping interactions formed between enhancer elements and genes that they regulate map within distinct chromosomal topological associating domains (TADs)^17^. To map candidate causal SNPs to TADs and identify patterns of local chromatin patterns, we analysed Hi-C data on the LCL cell line GM12878 (ref. 17), as a source of B-cell information (Supplementary Fig. 3). Looping chromatin interactions and TADs were shown at 6q21 (rs9372120), 8q24.21 (rs1948915), 9p21.3 (rs2811710) and 20q13.13 (rs6066835), involving a number of genes with biological relevance to MM development. With the limitations of cell line data from LCL, which may not fully reflect MM biology, we demonstrated with MM RNA-sequencing data that gene expression within the 6q21 and 9p21.3 TADs were tightly correlated (*P*<2.0 × 10^−5^), which is consistent with their co-regulation (Supplementary Table 17). Moreover, the region at 6q21 (rs9372120, *ATG5*) participates in intra-chromosome looping with the transcriptional repressor *PRDM1* (Supplementary Fig. 3b). Similarly, the 8q24.21 region of association defined by rs1948915, which contains *CCAT1* (colon cancer-associated transcript 1), interacts with *MYC* and distal upstream enhancer elements (Supplementary Fig. 3d).

To explore the epigenetic profile of association signals at each of the new MM risk loci, we used HaploReg and RegulomeDB to examine whether the sentinel SNPs and those in high LD (that is, *r*^2^>0.8 in the 1,000 Genomes EUR reference panel) annotate putative transcription factor (TF) binding or enhancer elements. We also assessed B-cell-specific chromatin dynamics using FANTOM5, which uses the pre-computed chromatin state data for multiple cell lines. HaploReg showed that the majority of MM-related SNPs were observed in regions of DNase hypersensitivity common across multiple cell lines. The protein motifs at these sites are for known TFs such as nuclear factor-κB, c-MYC, GATA, TCF4, POL24H8, CEBPB or POL2 (Supplementary Data 2). We examined for statistical evidence of enrichment in specific TF binding across the eight new and nine established risk loci using GM12878 data^18^. Although of borderline significance and hypothesis generating, after correction for the 90 TFs assayed, there was evidence for enrichment of SPI1 (alias PU.1), (*P*=0.0007, *P*_adjusted_=0.063), which regulates *PRDM1* and its downregulation is required for MM cell growth^19^. Collectively, these observations are compatible with the identified risk SNPs mapping within regions of active chromatin state, which have a role in the B-cell *cis*-regulatory network.

## Discussion

We have performed the largest GWAS of MM to date. We identified eight novel MM risk loci taking the total count to 17. Fully deciphering the functional impact of these SNP associations on MM development requires additional analyses. However, seven of the SNPs map intragenic to transcribed genes, which are relevant to MM or B-cell biology. Although a number of SNPs displayed an eQTL/meQTL in MM plasma cells, the absence of a relationship does not preclude the possibility of a subtle cumulative long-term relationship intrinsic to plasma cells or a predisposition through altered gene function in other cell types.

Studies in other cancers have shown that the multiple risk loci at 8q24.21 are enhancers interacting with *MYC*^20^,^21^. As deregulation of *MYC* is a feature of MM, it is plausible that the susceptibility to MM has a similar mechanistic basis. Indeed, *MYC* promotes *CCAT1* transcription by binding to its promoter, and in colorectal cancer the L-isoform of *CCAT1* has been shown to interact with the *MYC* promoter and distal upstream enhancer elements regulating *MYC* transcription^22^. We have previously shown the MM risk SNP at 7p15.3 influences expression of *CDCA7L*, a binding partner of p75 potentiating *MYC*-mediated transformation. In addition to local interactions with *CDKN2A/CDKN2B*, the 9p21.3 region encompassing SNP rs2811710 interacts with the genomic region containing *MTAP* (methylthioadenosine phosphorylase). *MTAP* plays a major role in polyamine metabolism and deletion of *MTAP* is common in cancer, being closely linked to homozygous deletion of *p16* (ref. 23).

*ATG5* at 6q21 is highly expressed in plasma cells and essential for autophagy and plasma cell survival^24^. Strikingly, the same locus also contains the transcriptional repressor *PRDM1* (formerly *BLIMP1*), which is key to the development of plasma cells from B cells and a determinant of plasma cell survival^25^. The RFWD3 protein is an E3 ubiquitin ligase that positively regulates p53 stability by forming an RFWD3–MDM2–p53 complex, thereby protecting p53 from degradation by MDM2-mediated polyubiquitination^26^. Variation at 16q23.1 defined with the correlated SNP rs4888262 (pairwise LD with rs7193541, *r*^2^=0.68, *D*'=1.0) has previously been shown to influence testicular cancer risk^27^, suggesting a common genetic and biological basis to both associations.

*JARID2* functions as a transcriptional repressor through recruitment of Polycomb repressive complex 2 and has recently been identified as a regulator of haematopoietic stem cell function^28^, and the 6p22.3-p21.31 region is commonly gained in MM tumours^29^. Inhibition of *JARID2* leads to loss of Polycomb binding and a reduction of histone H3 lysine-27 trimethylation levels on target genes. *SMARCD3* recruits BAF chromatin remodelling complexes to specific enhancers. Although there is currently no evidence to implicate the transcriptional repressors *JARID2* or *SMARCD3* in terms of somatic mutation in MM, multiple genes including *CDKN2A* and *TP53* are silenced by methylation in MM. Overexpression of histone methyltransferase and inactivating mutations in histone demethylase (*UTX*) typifies a subset of MM^30^ and our findings add to the impact of chromatin remodelling genes on MM.

We have previously shown an association for MM at *ULK4*, a key regulator of mammalian target of rapamycin-mediated autophagy^4^. We now suggest a more extensive set of associations involving *ATG5* and *WAC*, and by virtue of the role of MYC in autophagy^31^, *CCAT1*, *CDCA7L*, *DNMT3A* and *CBX7*. Collectively, these data invoke deregulation of DNA methylation, telomere length, differentiation and autophagy, and immunoglobulin production as determinants of MM susceptibility.

Our findings provide further evidence for an inherited genetic susceptibility to MM. However, further studies are necessary to understand the biology behind these risk variants. We estimate that the currently identified risk SNPs for MM account for 20% of the heritable risk attributable to all common variation; hence, further GWAS-based studies in concert with functional analyses should lead to additional insights into MM biology. Importantly, such studies may inform the development of new therapeutic agents^32^,^33^.

## Methods

### Ethics

Collection of patient samples and associated clinico-pathological information was undertaken with written informed consent and relevant ethical review board approval at respective study centres in accordance with the tenets of the Declaration of Helsinki, specifically for the Myeloma-IX trial by the Medical Research Council (MRC) Leukaemia Data Monitoring and Ethics committee (MREC 02/8/95, ISRCTN68454111), the Myeloma-XI trial by the Oxfordshire Research Ethics Committee (MREC 17/09/09, ISRCTN49407852), HOVON65/GMMG-HD4 (ISRCTN 644552890; METC 13/01/2015), HOVON87/NMSG18 (EudraCTnr 2007-004007-34, METC 20/11/2008), HOVON95/EMN02 (EudraCTnr 2009-017903-28, METC 04/11/10), University of Heidelberg Ethical Commission (229/2003, S-337/2009, AFmu-119/2010), University of Arkansas for Medical Sciences Institutional Review Board (IRB 202077), Lund University Ethical Review Board (2013/54) and Icelandic Data Protection Authority (2,001,010,157 and National Bioethics Committee 01/015).

### Genome-wide association studies

The diagnosis of MM (ICD-10 C90.0) was established in accordance with World Health Organization guidelines. All samples from patients for genotyping were obtained before treatment or at presentation. The meta-analysis was based on GWAS conducted in the Netherlands, the United Kingdom, Germany, Sweden/Norway, the United States and Iceland (Supplementary Tables 1 and 2).

The Dutch GWAS consisted of 608 cases (316 male). The cases were ascertained from three clinical trials: HOVON65/GMMG-HD4 ISRCTN64455289 (restricted to Dutch cases; *n*=158), HOVON87/NMSG18 (*n*=292) and HOVON95/EMN02 (*n*=105) (ISRCTN64455289: GMMG-HD4 http://www.isrctn.com/search?q=ISRCTN64455289, HOVON87/NMSG18; HOVON87/NMSG18 https://www.clinicaltrialsregister.eu/ctr-search/trial/2007-004007-34/BE and HOVON95/EMN02 https://www.clinicaltrialsregister.eu/ctr-search/trial/2009-017903-28/AT). DNA was extracted from venous blood samples and genotyped using Illumina Human OmniExpress-12 v1.0 arrays (Illumina, San Diego, USA). For controls, we used the B-PROOF data set (B-vitamins for the prevention of osteoporotic fractures). Controls were genotyped using Illumina OmniEpress Exome-8v1-1 arrays^34^.

The UK GWAS^2^ comprised 2,329 cases (1,060 male (post quality control (QC)); mean age at diagnosis: 64 years) recruited through the UK MRC Myeloma-IX and Myeloma-XI trials (ISRCTN68454111: Myeloma IX http://www.isrctn.com/search?q=ISRCTN68454111 and ISRCTN49407852: Myeloma XI http://www.isrctn.com/search?q=ISRCTN49407852). DNA was extracted from EDTA-venous blood samples (90% before chemotherapy) and genotyped using Illumina Human OmniExpress-12 v1.0 arrays (Illumina). For controls, we used publicly accessible data generated by the Wellcome Trust Case Control Consortium from the 1958 Birth Cohort (58C; also known as the National Child Development Study) and National Blood Service. Genotyping of controls was conducted using Illumina Human 1-2M-Duo Custon_v1 Array chips (www.wtccc.org.uk).

The German GWAS^2^ comprised 1,512 cases (867 male (post QC); mean age at diagnosis: 59 years) recruited by the German-Speaking Multiple Myeloma Multicenter Study Group (GMMG) coordinated by the University Clinic, Heidelberg (ISRCTN06413384: GMMG-HD3 http://www.isrctn.com/search?q=ISRCTN06413384; ISRCTN64455289: GMMG-HD4 http://www.isrctn.com/search?q=ISRCTN64455289; and ISRCTN05745813: GMMG-HD5 http://www.isrctn.com/search?q=ISRCTN05745813). DNA was prepared from EDTA-venous blood or CD138-negative bone marrow cells (<1% tumour contamination). Genotyping was performed using Illumina Human OmniExpress-12 v1.0 arrays (Illumina). For controls, we used genotype data on 2,107 healthy individuals, enroled into the Heinz Nixdorf Recall (HNR) study genotyped using either Illumina HumanOmni1-Quad_v1 or 1428 OmniExpress-12 v1.0 arrays.

The Swedish/Norwegian GWAS^5^ was based on 1,668 and 157 MM patients from the Swedish National Myeloma Biobank (Skåne University Hospital, Lund, Sweden) and the Norwegian Biobank for Myeloma (Trondheim, Norway), respectively. Genotyping was performed using Illumina Human OmniExpress-Exome arrays (Illumina). Control genotypes on 10,704 individuals were obtained from previously published studies of schizophrenia and TWINGENE^5^.

The USA GWAS^7^ comprised 1,076 newly diagnosed patients treated at the UAMS Myeloma Institute for Research and Therapy (NCT00083551: Total therapy II https://clinicaltrials.gov/ct2/show/NCT00083551; NCT00081939: Total therapy III https://clinicaltrials.gov/ct2/show/NCT00081939; NCT00572169: Total therapy 3B https://clinicaltrials.gov/ct2/show/NCT00572169; and NCT00734877: Total therapy 4 https://clinicaltrials.gov/ct2/show/NCT00734877). DNA was isolated from peripheral blood samples collected from patients after granulocyte–colony-stimulating factor mobilization of stem cells. Genotyping was performed using Illumina Human OmniExpress-12 v1.0 arrays and OmniExpress arrays (Illumina)^7^. Genotype data from 2,234 healthy individuals enroled into the Cancer Genetic Markers of Susceptibility studies served as a source of controls.

The Icelandic GWAS comprised 480 MM cases identified from the nationwide Icelandic Cancer Registry^5^. Samples were genotyped using Illumina microarrays^5^.

### Analysis of GWAS

The Swedish/Norwegian GWAS has been previously published in its entirety with a full description of QC^5^. Adopting the same standard, quality-control measures were applied to the UK, German, US and the Netherlands GWAS. Specifically, we excluded individuals with low call rate (<95%) and those found to have non-European ancestry on the basis of HapMap version 2 CEU, JPT/CHB and YRI population reference data (Supplementary Fig. 4). For first-degree relative pairs, we excluded the control or the individual with the lower call rate. SNPs with a call rate <95% were excluded as were those with a MAF<0.01 or displaying significant deviation from Hardy–Weinberg equilibrium (that is, *P*<10^−5^). Post QC, the 5 GWAS provided genotype data on 6,839 cases and 22,221 controls. GWAS data were imputed for all scans for >10 million SNPs using 1,000 Genomes Project (phase 1 integrated release 3, March 2012)^8^ and UK10K data (ALSAPAC, EGAS00001000090/EGAD00001000195 and TwinsUK EGAS00001000108/EGAS00001000194 studies only)^9^ as reference in conjunction with IMPUTE2 v2.3 software^35^ (Supplementary Tables 1 and 2). Imputation was conducted separately for each scan and each GWAS was pruned to a common set of SNPs between cases and controls. We pre-set thresholds for imputation quality, to retain potential risk variants with MAF>0.005 for validation. Specifically, we excluded poorly imputed SNPs (that is, information measure *I*s <0.80). Test of association between imputed SNPs and MM was performed using logistic regression using SNPTESTv2.5.2 (ref. 36). The adequacy of the case–control matching was formally evaluated using quantile–quantile plots of test statistics (Supplementary Fig. 1). The inflation factor *λ* was based on the 90% least-significant SNPs^37^. Where appropriate, principle components (zero for UK, five for Sweden/Norway, two for Germany, zero for USA and zero for the Netherlands), generated using common SNPs, were included to limit the effects of cryptic population stratification. Eigenvectors for the GWAS data sets were inferred using smartpca (part of EIGENSOFT^38^) by merging cases and controls with Phase II HapMap samples.

For the Icelandic GWAS, SNP genotypes were phased using a long-range method based on whole genome sequence data on 2,636 Icelanders. Sequence variants (35.5 million) were then imputed into 104,220 Icelanders, which had been genotyped using Illumina chips. We corrected for familial relatedness by genomic control dividing the *χ*^2^-statistic by 1.04.

### Meta-analysis

We performed association testing in the discovery sets separately and then combined the results for 12.4 million variants. We assessed the fidelity of imputation through the concordance between imputed and directly genotyped SNPs in a subset of GWAS samples (Supplementary Tables 6 and 7). Meta-analysis was undertaken using the inverse-variance approach under a fixed-effects model implemented in META v1.6 (ref. 39). Cochran's *Q*-statistic was calculated, to test for heterogeneity, and the *I*^2^ statistic measured, to quantify the proportion of the total variation due to heterogeneity^40^. Meta-analysis summary statistics and LD correlations from a reference panel of 1,000 Genomes Project combined with UK10K, we used GCTA^41^ to perform conditional association analysis. Association statistics were calculated for all SNPs conditioning on the top SNP in each loci showing genome-wide significance. This is performed in a step-wise manner.

### Replication genotyping

To validate promising associations, we analysed four case–control series from the United Kingdom, Germany, Denmark and Sweden/Norway.

The UK replication comprised 812 MM cases (412 male) ascertained through the UK MRC Myeloma-IX (*n*=95) and XI trials (*n*=717). Controls comprised 1,110 healthy individuals with self-reported European ancestry (420 male, aged 18–69 years) with no personal history of malignancy ascertained through GEnetic Lung CAncer Predisposition Study (*n*=536) (ref. 42) and National Study of Colorectal Cancer Genetics (*n*=574) (ref. 43). All cases and controls were UK residents.

The German replication series comprised 1,149 cases collected by the German Myeloma Study Group (Deutsche Studiengruppe Multiples Myelom (DSMM)), GMMG, University Clinic, Heidelberg, and University Clinic, Ulm (676 male, mean age at diagnosis 57.6 years, s.d. 9.8). Controls comprised of 1,582 healthy German blood donors recruited between 2004 and 2007 by the Institute of Transfusion Medicine and Immunology, University of Mannheim, Germany (885 male, mean age 55.8 years, s.d. 10.0).

The Swedish/Norway and Danish replication series comprised 223 MM cases from the Swedish National Myeloma Biobank and 363 MM cases from the University Hospital of Copenhagen. As controls for these respective replication sets, we analysed 1,285 Swedish blood donors and 826 individuals from Denmark and Skåne County, Sweden (the southernmost part of Sweden adjacent to Denmark).

Replication genotyping was performed using allele-specific PCR KASPar chemistry (LGC, Hertfordshire, UK; UK replication series). Primers, probes and conditions used are available on request. Call rates for SNP genotypes were >95% in each of the replication series. The quality of genotyping in all assays was assessed by measuring 1–10% duplicates (showing a concordance of >99%) and at least two negative controls for each centre. Technical artefacts were excluded by cross-platform validation of 96 samples and sequencing of a set of 96 randomly selected samples from each case and control series confirmed genotyping accuracy. Concordance of >99% demonstrated robust performance.

### Translocation detection and mutation analysis

Karotyping was used for cytogenetic studies of MM cells and standard criteria for the definition of a clone were applied. Fluorescence *in situ* hybridization and ploidy classification of UK samples was conducted using the methodologies previously described^44^. Fluorescence *in situ* hybridization and ploidy classification of German samples was performed as previously described^45^. The XL IGH Break Apart probe (MetaSystems, Altlussheim Germany) was used to detect any IGH translocation in German samples. Logistic regression in case-only analyses was used to assess tumour karyotype . The frequency of somatic mutation in genes annotated by GWAS signals was derived from tumour whole-exome sequencing of 463 Myeloma XI trial patients^46^.

### Association between genotype and patient outcome

To examine the relationship between SNP genotype and patient outcome, we analysed GWAS data on four of the patient cohorts^2^,^3^,^4^,^7^, specifically (i) 1,165 cases from the UK MRC Myeloma-IX trial (UK-GWAS); (ii) 877 MM cases from the UK MRC Myeloma-XI trial (UK-GWAS); (iii) 511 of the patients recruited to the German GWAS; and (iv) 703 MM cases in the UAMS Myeloma Institute for Research and Therapy GWAS (USA GWAS)^7^. Clinical trial information on these patients has been previously reported^47^,^48^,^49^,^50^. The primary analysis end point was myeloma-specific overall survival and analysis was performed as previously described^51^. Cox regression analysis was used to derive genotype-specific hazard ratio and associated 95% confidence intervals. Meta-analysis was performed under a fixed-effects model (Supplementary Table 15).

### eQTL analysis

We performed an eQTL analyses using Affymetrix Human Genome U133 2.0 Plus Array data for plasma cells from 183 MRC Myeloma IX trial patients^29^, 658 Heidelberg patients and 608 US patients as recently described. Briefly, GER, UK and US data were separately pre-processed and analysed using a Bayesian approach to probabilistic estimation of expression residuals to infer broad variance components, thus accounting for hidden determinants influencing global expression such as copy number, translocation status and batch effects^52^. The association between genotype of the sentinel variant and gene expression of genes within 500 Kb either side was evaluated based on the significance of linear regression coefficients. We pooled data from each study under a fixed-effects model controlling for false discovery rate (FDR) calling significant associations with a FDR≤0.05. In addition, we queried publicly available eQTL messenger RNA expression data using MuTHER and the Blood eQTL browser. MuTHER contains expression data on LCLs, skin and adipose tissue from 856 healthy twins^53^. The Blood eQTL browser contains expression data from 5,311 non-transformed peripheral blood samples^54^.

### meQTL analysis

We performed *cis*-meQTL analysis using Illumina 450K methylation array data on plasma cells from 384 MRC Myeloma XI trial patients. As with analysis of MM expression (eQTL) data, we inferred hidden determinants influencing global methylation. The genetic association was tested under an additive model between each SNP and each normalized methylation probe, adjusting for plate and methylation-based principal component analysis score. Controlling for a FDR of 0.05 across the 338,456 methylation traits required a *P*-value for association to be <4.0 × 10^−5^.

### ENCODE and chromatin state dynamics

Risk SNPs and their proxies (that is, *r*^2^>0.8 in the 1,000 Genomes EUR reference panel) were annotated for putative functional effect using HaploReg v3 (ref. 55), RegulomeDB^56^ and SeattleSeq^57^ annotation. These servers make use of data from ENCODE^58^, genomic evolutionary rate profiling^59^ conservation metrics, combined annotation dependent depletion scores^60^ and PolyPhen scores^61^. We examined for an overlap of associated SNPs with predicted enhancers using the FANTOM5 enhancer atlas^62^and searched for overlap with ‘super-enhancer' regions using data from Hnisz *et al.*^63^, restricting our analysis to GM12878.

To formally examine for enrichment in specific TF binding across risk loci, we adopted the method of Gaulton *et al.*^18^ Briefly, for each risk locus we derived a credible set of SNPs with a 99% probability of containing the causal SNP; posterior probability for each SNP being computed from its Bayes factor. SNPs were ranked by their posterior probability and included so that the cumulative posterior probability for association was >0.99. Binding sites for 90 TF in GM12878 were obtained from ENCODE. For each TF the total posterior probability over all credible set SNPs overlapping all binding sites was calculated. A null distribution was generated by randomly relocating each binding site up to 100 kb from its original location. For these perturbed sites, the total posterior probability over all overlapping SNPs was calculated. This process was repeated 10,000 times and enrichment *P*-values calculated as the fraction of permutations where the total posterior probability was greater than for the unperturbed binding sites.

### Hi-C data and definition of topological domains at risk loci

Hi-C data was used to map the candidate causal SNPs to chromosomal TADs and identify patterns of relevant, local chromatin interactions. We made use of publicly available raw Hi-C data on GM12878 cells^17^. Valid Hi-C pairs were generated aligning raw reads to the reference genome using Burrows-Wheeler alignment (BWA), matching pairs of reads and filtering for biases. *Bona fide* Hi-C ditags were allocated to a contact matrix, with a predefined, uniform resolution of 5 kb. We corrected for experimental bias using the matrix balancing approach^64^. We inferred TADs from the contact matrix by means of the arrowhead algorithm for domain detection as previously proposed.

To investigate whether genes within TADs are co-regulated, we obtained RNAseq transcript counts from 66 MM cell lines from the Keat's lab Data Repository (http://www.keatslab.org/data-repository)^65^. We performed pairwise correlation by calculating the Pearson's product–moment correlation coefficient of the transcript counts for all pairs of genes within respective TADs.

### Heritability analysis

We used Genome-wide Complex Trait Analysis to estimate the polygenic variance ascribable to all genotyped and imputed GWAS SNPs simultaneously for the UK and German GWAS^41^,^66^,^67^. SNPs were excluded based on low MAF, poor imputation and poor HWE. Principal components were included as covariates in the heritability analysis of the German data. As previously advocated when calculating the heritability of a disease such as cancer we used the lifetime risk^68^,^69^, which for MM is estimated to be 0.007 for the UK population (http://www.cancerresearchuk.org/cancer-info/cancerstats/types/myeloma/incidence/uk-multiple-myeloma-incidence-statistics#Lifetime) and 0.006 for the German population. We estimated the heritability explained by risk SNPs identified by GWAS as located within regions associated with MM. Meta-analysis of heritability estimates from UK and German GWAS data sets was performed under a standard fixed-effects model.

### Data availability

SNP genotyping data that support the findings of this study have been deposited in Gene Expression Omnibus with accession codes GSE21349, GSE19784, GSE24080, GSE2658 and GSE15695; in the European Genome-phenome Archive (EGA) with accession code EGAS00000000001; in the European Bioinformatics Institute (Part of the European Molecular Biology Laboratory) (EMBL-EBI) with accession code E-MTAB-362 and E-TABM-1138; and in the database of Genotypes and Phenotypes (dbGaP) with accession code phs000207.v1.p1.

Expression data that support the findings of this study have been deposited in GEO with accession codes GSE21349, GSE2658, GSE31161 and EMBL-EBI with accession code E-MTAB-2299.

Whole-exome sequence data that support the findings of this study have been deposited in EGA with accession code EGAS00001001147.

Transcription profiling data from MuTHer studies that support the findings of this study have been deposited in EMBL-EBI with accession code E-TABM-1140. Data from Blood eQTL have been deposited in EMBL-EBI with accession codes E-TABM-1036, E-MTAB-945 and E-MTAB-1708.

The remaining data are contained within the paper and Supplementary Files or available from the author upon request.

## Additional information

**How to cite this article:** Mitchell, J. S. *et al.* Genome-wide association study identifies multiple susceptibility loci for multiple myeloma. *Nat. Commun.* 7:12050 doi: 10.1038/ncomms12050 (2016).

## Supplementary Material

Supplementary Information Supplementary Figures 1-4, Supplementary Tables 1-17 and Supplementary References.

Supplementary Data 1 eQTL and meQTL analysis.

Supplementary Data 2 Epigenetic annotation of genome-wide significant SNPs.

## Figures and Tables

**Table 1:** Summary results for SNPs associated with multiple myeloma risk.

**Location**	**SNP**	**Position (bp)**	**Risk allele**	**RAF**	**Data set**	**OR**	***P*****-value**
6p22.3	rs34229995	15,244,018	G	0.029	Discovery	1.40	1.76 × 10^−8^
					Replication	1.19	0.214
					Combined	**1.37**	**1.31 × 10**^**−8**^
						*P*_het_=0.50	*I*^2^=0%
							
6q21	rs9372120	106,667,535	G	0.218	Discovery	1.20	8.72 × 10^−14^
					Replication	1.12	0.0147
					Combined	**1.18**	**9.09 × 10**^**−15**^
						*P*_het_=0.93	*I*^2^=0%
							
7q36.1	rs7781265	150,950,940	T	0.125	Discovery	1.20	1.82 × 10^−7^
					Replication	1.15	0.0136
					Combined	**1.19**	**9.71 × 10**^**−9**^
						*P*_het_=0.24	*I*^2^=23%
							
8q24.21	rs1948915	128,222,421	C	0.345	Discovery	1.14	3.14 × 10^−10^
					Replication	1.09	0.0283
					Combined	**1.13**	**4.20 × 10**^**−11**^
						*P*_het_=0.34	*I*^2^=11%
							
9p21.3	rs2811710	21,991,923	G	0.657	Discovery	1.14	6.50 × 10^−10^
					Replication	1.18	4.02 × 10^−5^
					Combined	**1.15**	**1.72 × 10**^**−13**^
						*P*_het_=0.97	*I*^2^=0%
							
10p12.1	rs2790457	28,856,819	G	0.739	Discovery	1.12	8.44 × 10^−7^
					Replication	1.13	6.18 × 10^−3^
					Combined	**1.12**	**1.77 × 10**^**−8**^
						*P*_het_=0.94	*I*^2^=0%
							
16q23.1	rs7193541	74,664,743	T	0.585	Discovery	1.12	1.14 × 10^−8^
					Replication	1.17	4.79 × 10^−4^
					Combined	**1.13**	**5.00 × 10**^**−12**^
						*P*_het_=0.15	*I*^2^=35%
							
20q13.13	rs6066835	47,355,009	C	0.083	Discovery	1.24	1.16 × 10^−9^
					Replication	1.35	1.36 × 10^−5^
					Combined	**1.26**	**1.36 × 10**^**−13**^
						*P*_het_=0.072	*I*^2^=43%
							

*I*^2^, proportion of the total variation due to heterogeneity; OR, odds ratio; *P*_het_, *P*-value for heterogeneity; RAF, risk allele frequency; SNP, single-nucleotide polymorphism.

RAF is risk allele frequency across all cases and controls in the discovery set, where the risk allele is the allele corresponding to the estimated OR. Positions are based on NCBI build 37 of the human genome.
