# Supplementary material for: Genome-wide association study identifies multiple susceptibility loci for multiple myeloma
Source: Nat Commun. 2016 Jul 1;7:12050. doi: 10.1038/ncomms12050 (PMC4932178; doi:10.1038/ncomms12050)
Supplement: Supplementary Information — Supplementary Figures 1-4, Supplementary Tables 1-17 and Supplementary References. [file ncomms12050-s1.pdf]

a)

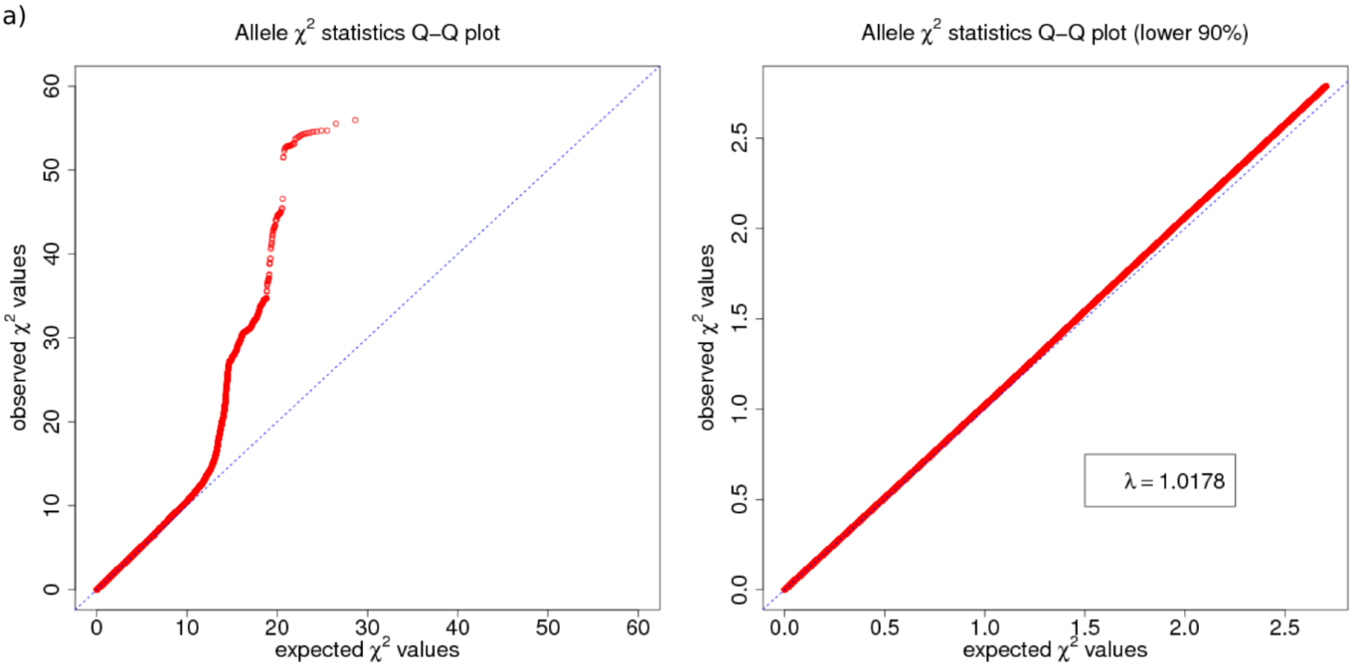
Allele χ2 statistics Q–Q plot
observed χ2 values
expected χ2 values
0
10
20
30
40
50
60
Allele χ2 statistics Q–Q plot (lower 90%)
0.0
0.5
1.0
1.5
2.0
2.5
λ = 1.0178

b)

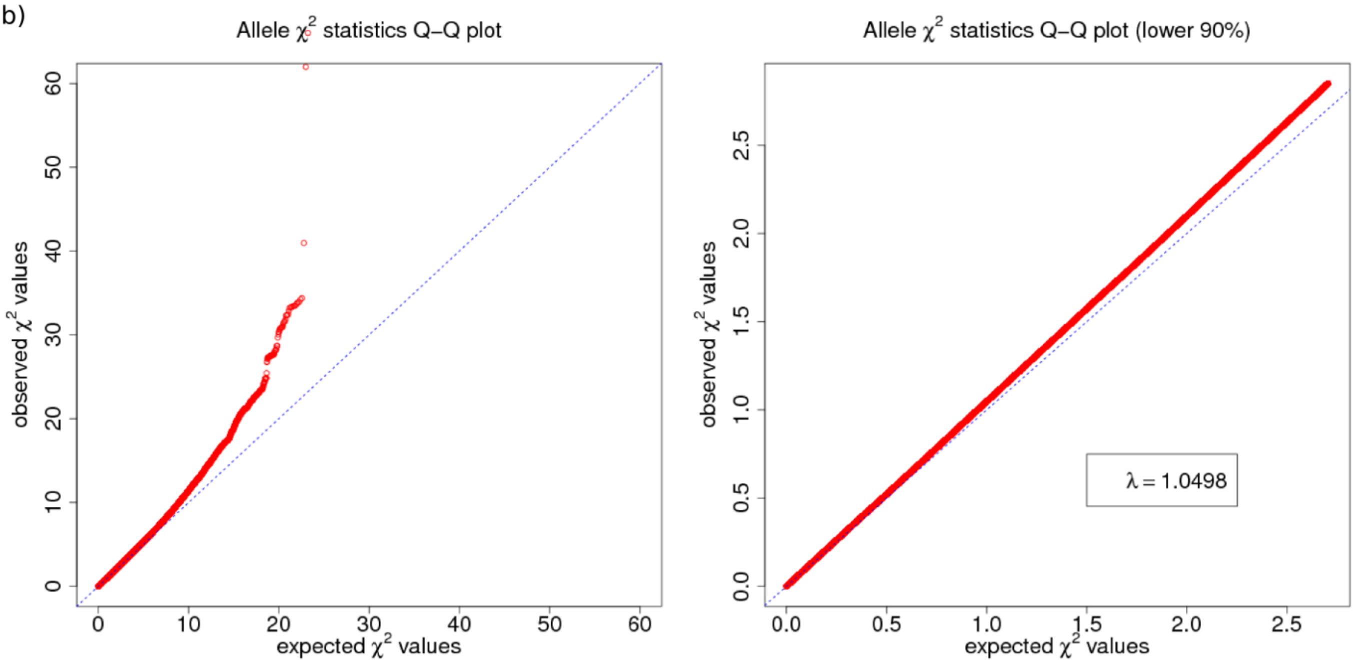
Allele χ2 statistics Q–Q plot
observed χ2 values
expected χ2 values
0
10
20
30
40
50
60
Allele χ2 statistics Q–Q plot (lower 90%)
0.0
0.5
1.0
1.5
2.0
2.5
λ = 1.0498

c)

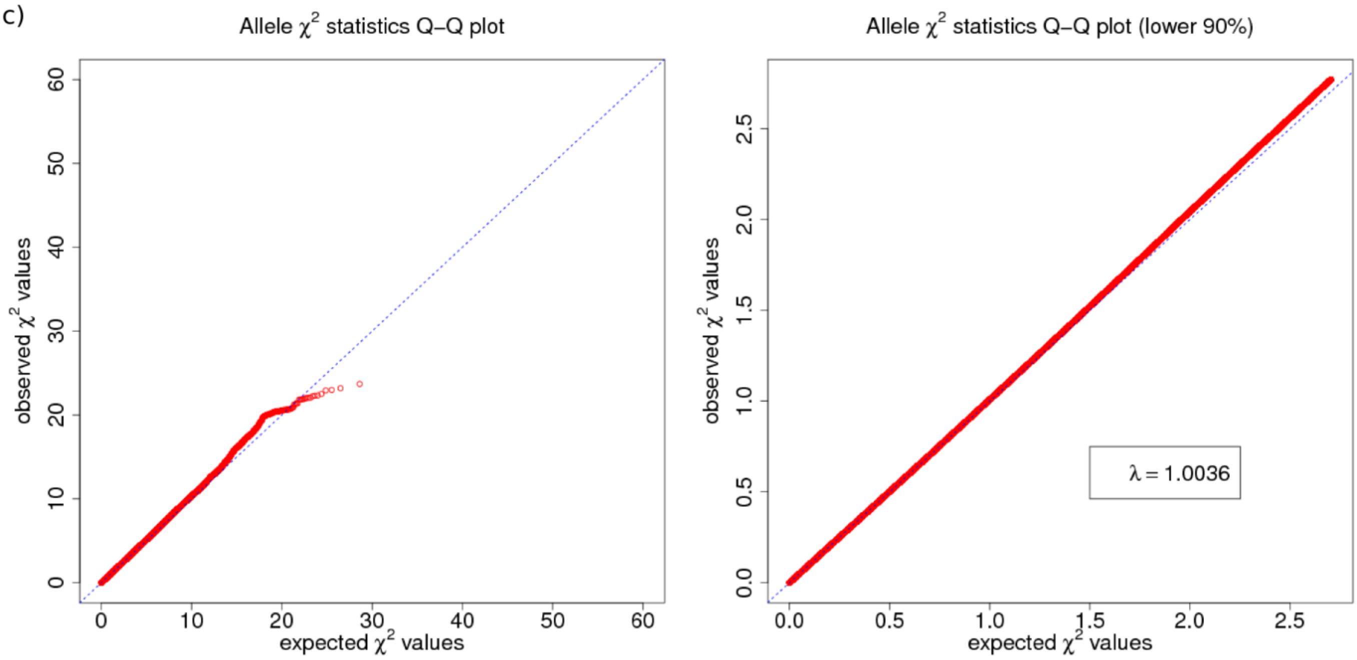

Allele χ^2 statistics Q–Q plot
observed χ^2 values
expected χ^2 values
Allele χ^2 statistics Q–Q plot (lower 90%)
$\lambda = 1.0036$

d)

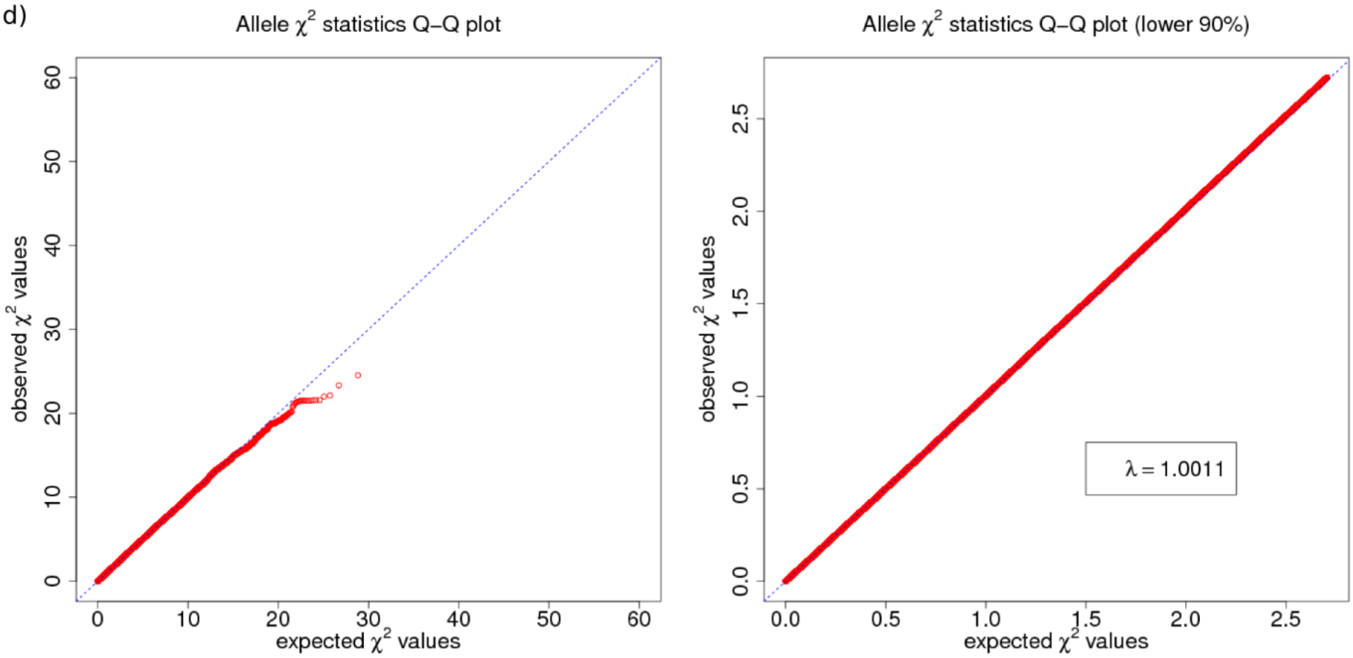

Allele χ^2 statistics Q–Q plot
observed χ^2 values
expected χ^2 values
Allele χ^2 statistics Q–Q plot (lower 90%)
$\lambda = 1.0011$

e)

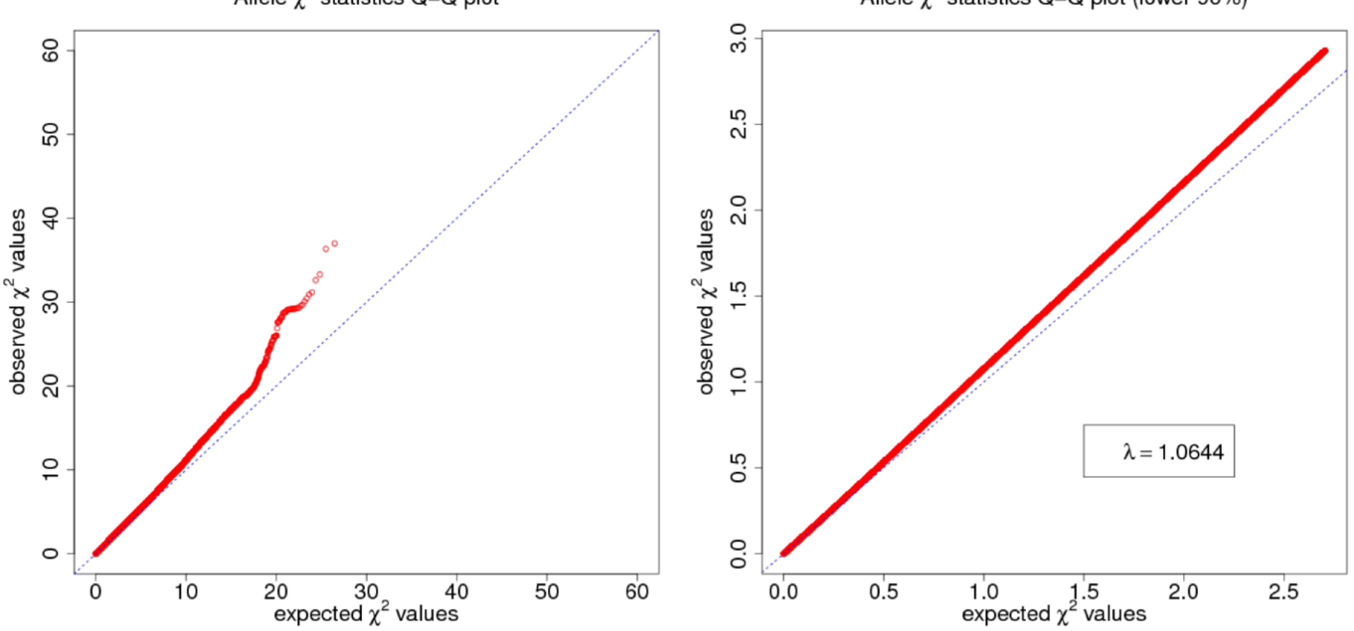

f)

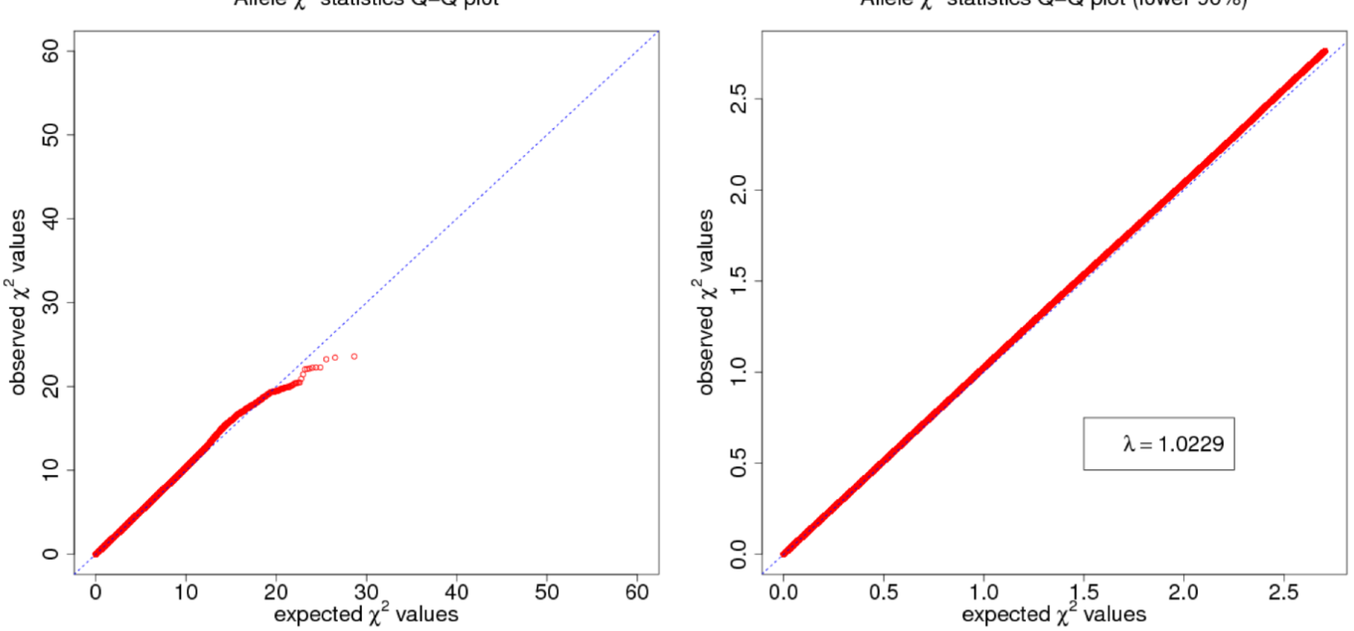

Supplementary Figure 1: Quantile-Quantile (Q-Q) plots of observed and expected χ^2 values of association between SNP genotype and risk of multiple myeloma after imputation. a) UK, b) Sweden/Norway, c) Germany, d) Iceland, e) USA and f) Netherlands. The blue line represents the null hypothesis of no true association.

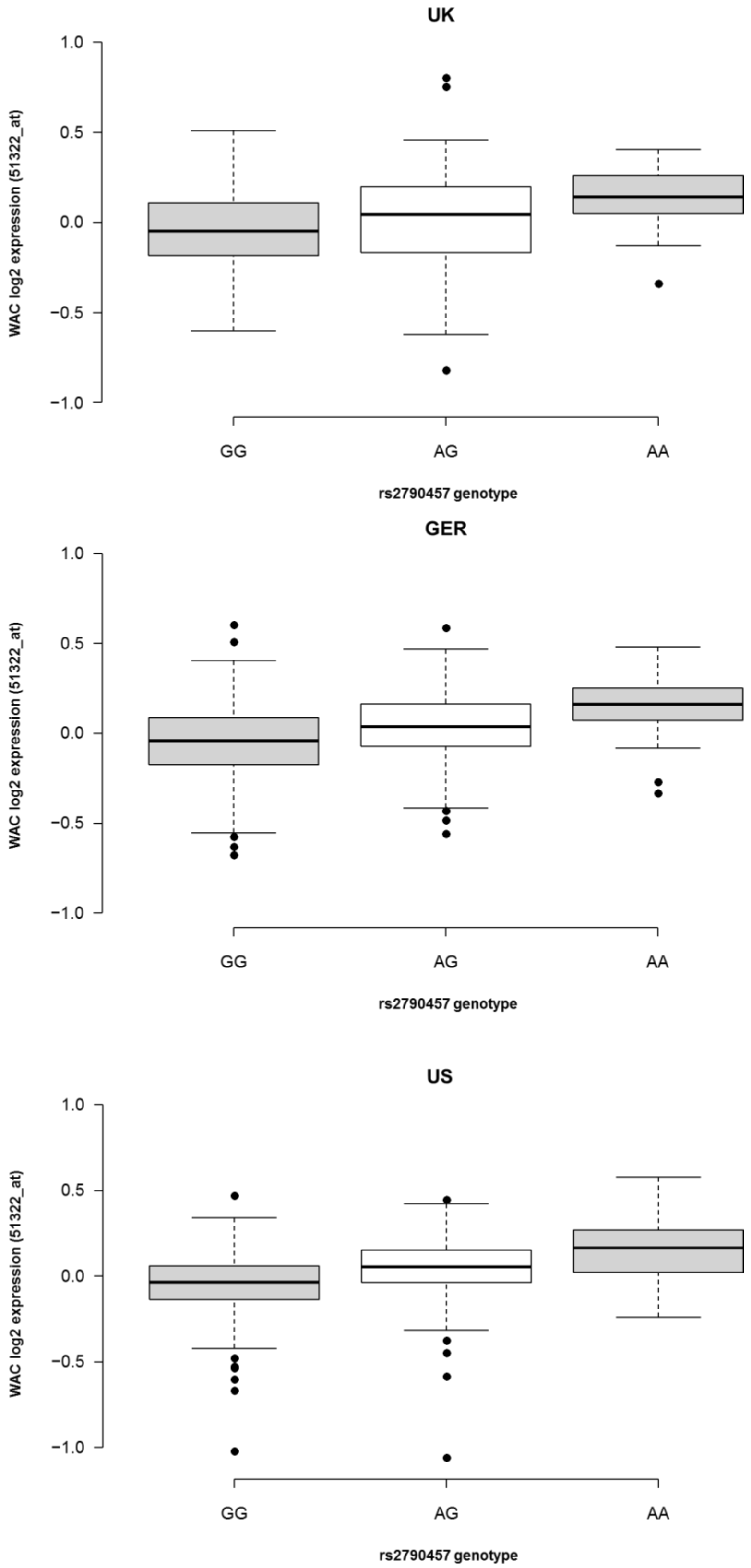
UK
GER
US
WAC log2 expression (51322_at)
rs2790457 genotype
GG
AG
AA
1.0
0.5
0.0
−0.5
−1.0

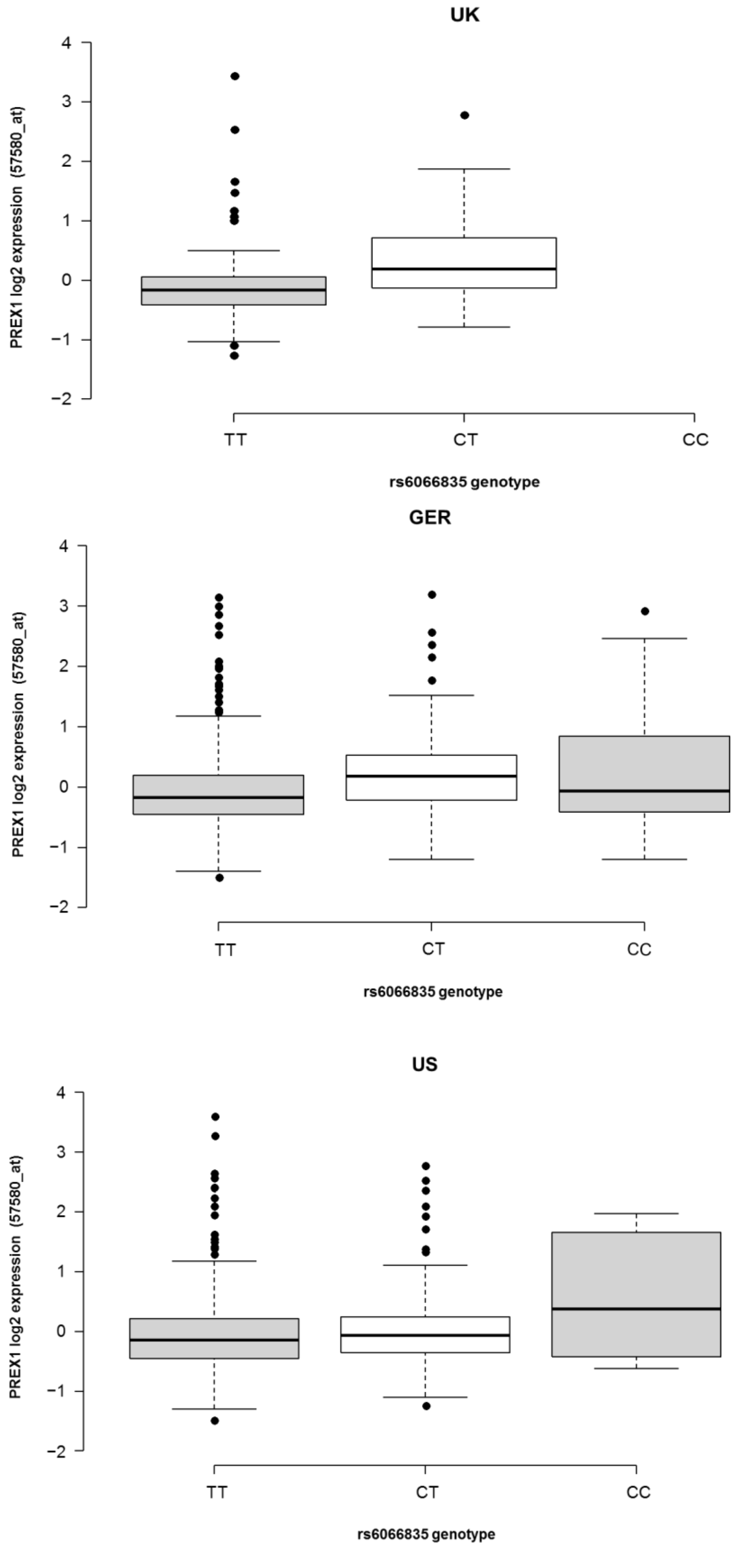

Supplementary Figure 2: eQTL boxplots. Relationship between rs2790457 (10p12.1) genotype and *WAC* expression, and rs6066835 (20q13.13) and *PREX1* in CD138+ selected plasma cells from 658 German, 183 UK and 608 US myeloma patients. The central line in each box indicates the median; the bottom and top lines of the box are the 25th and 75th percentiles; whiskers extend 1.5 times from the 25th and 75th percentiles.

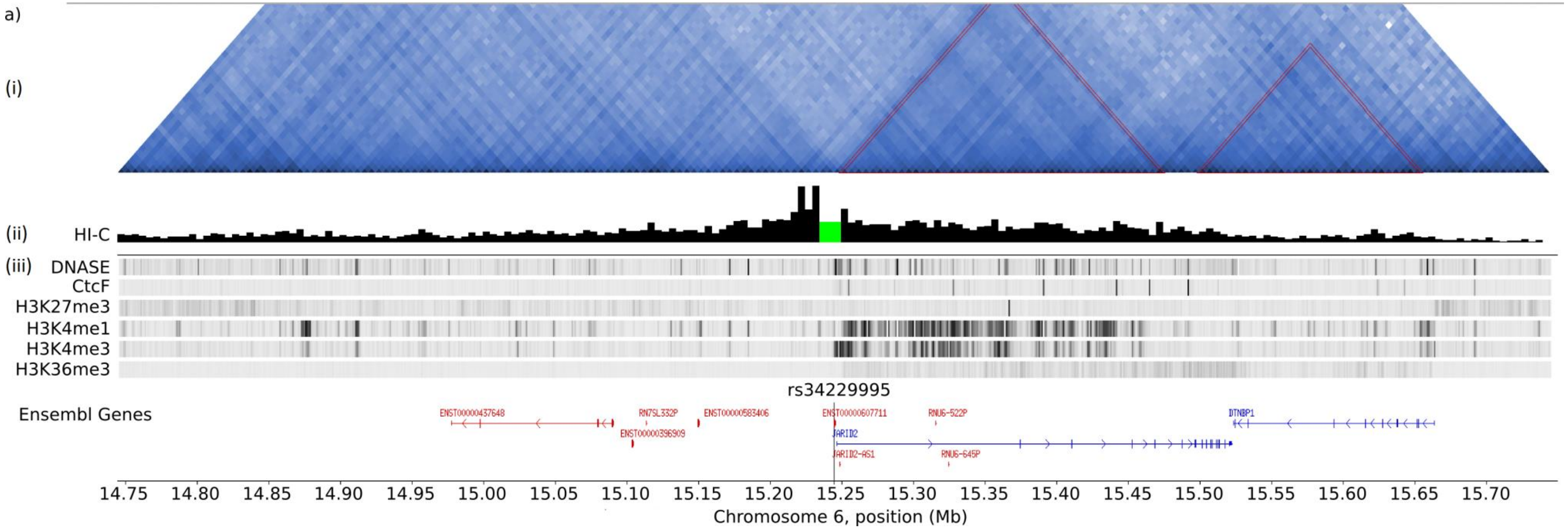

a)
(i)
(ii)
HI-C
(iii)
DNASE
CtcF
H3K27me3
H3K4me1
H3K4me3
H3K36me3
rs34229995
Ensembl Genes
ENST00000437648
RN7SL332P
ENST00000583406
ENST00000607711
RNU6-522P
DTNBP1
ENST00000396909
JARID2
JARID2-AS1
RNU6-645P
14.75 14.80 14.85 14.90 14.95 15.00 15.05 15.10 15.15 15.20 15.25 15.30 15.35 15.40 15.45 15.50 15.55 15.60 15.65 15.70
Chromosome 6, position (Mb)

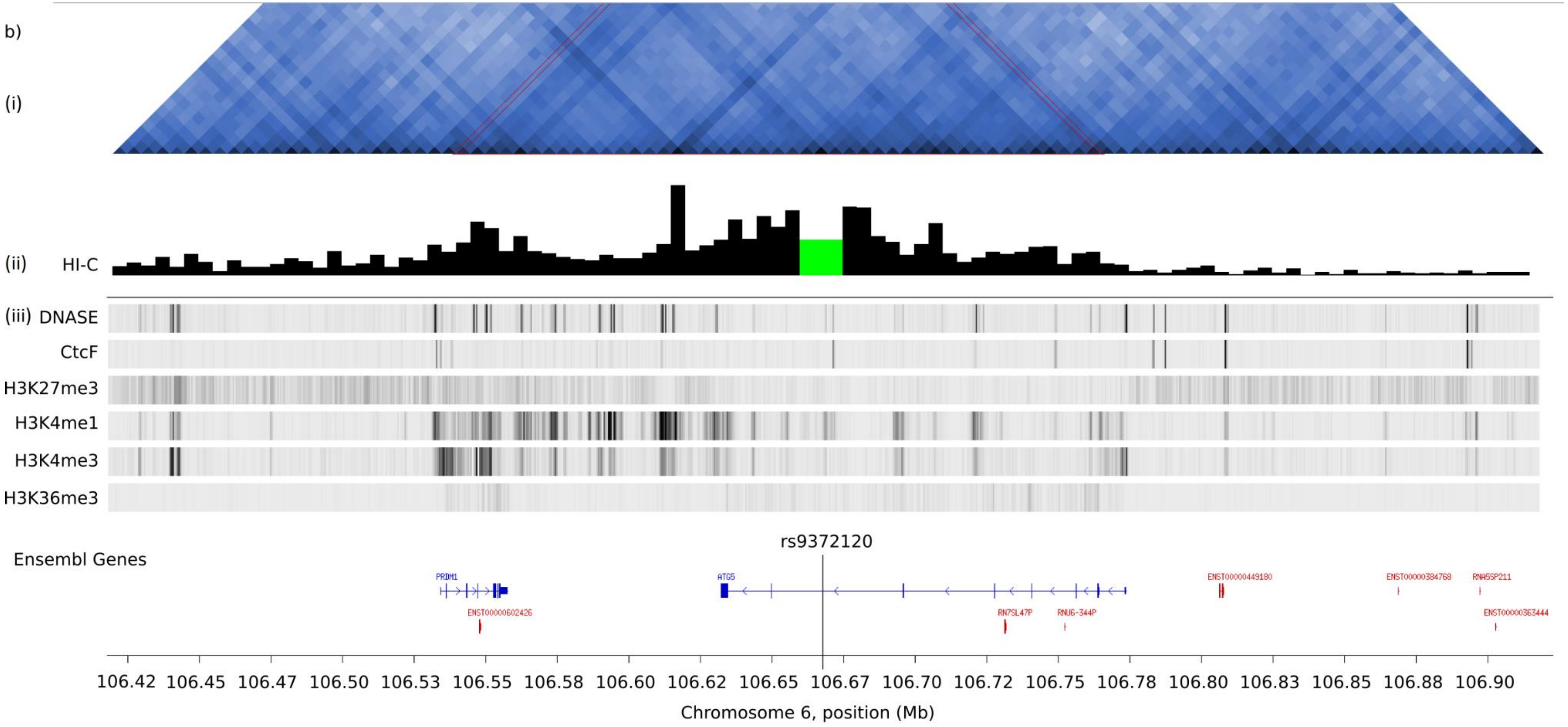

b)
(i)
(ii)
HI-C
(iii) DNASE
CtcF
H3K27me3
H3K4me1
H3K4me3
H3K36me3
Ensembl Genes
rs9372120
PRDM1
ATG5
ENST00000602426
RN7SL47P
RNU6-344P
ENST00000449180
ENST00000384768
RNA5SP211
ENST00000363444
106.42 106.45 106.47 106.50 106.53 106.55 106.58 106.60 106.62 106.65 106.67 106.70 106.72 106.75 106.78 106.80 106.83 106.85 106.88 106.90
Chromosome 6, position (Mb)

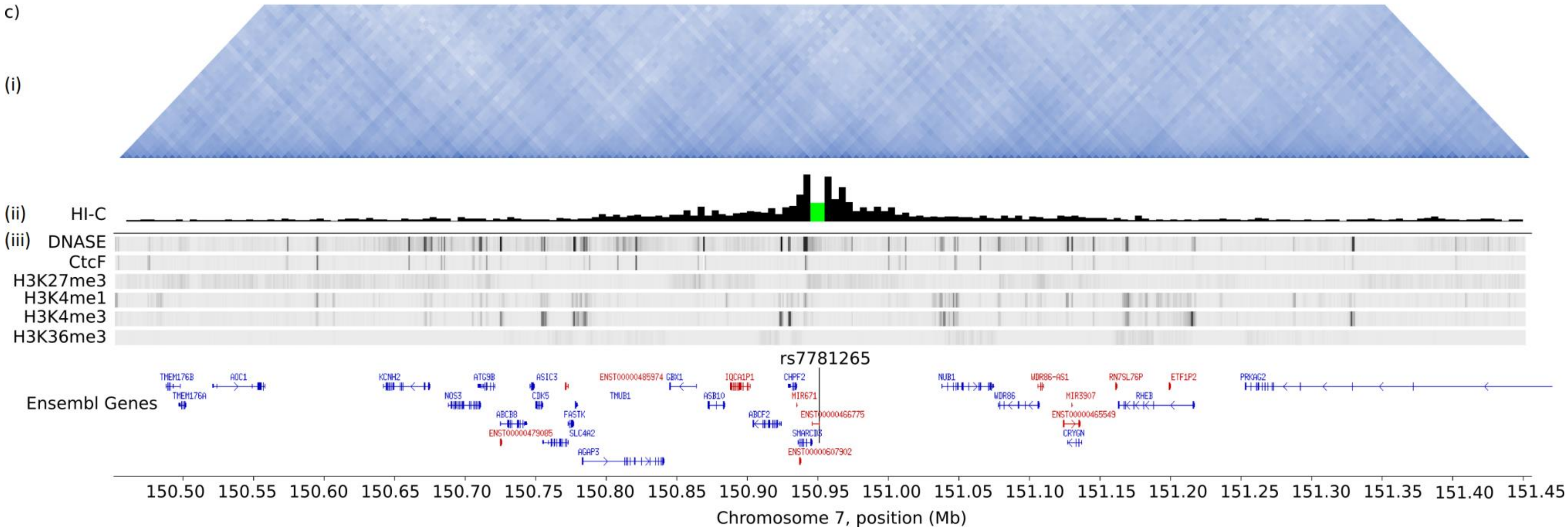

c)
(i)
(ii)
HI-C
(iii)
DNASE
CtcF
H3K27me3
H3K4me1
H3K4me3
H3K36me3
rs7781265
Ensembl Genes
TMEM176B
AOC1
TMEM176A
KCNH2
NOS3
ATG9B
ABCB8
ASIC3
CDK5
FASTK
SLC4A2
AGAP3
TMUB1
GBX1
ASB10
IQCA1P1
ABCF2
CHPF2
MIR671
SMARCD3
NUB1
WDR86
WDR86-AS1
MIR3907
CRYGN
RN7SL76P
RHEB
ETF1P2
PRKAG2
150.50
150.55
150.60
150.65
150.70
150.75
150.80
150.85
150.90
150.95
151.00
151.05
151.10
151.15
151.20
151.25
151.30
151.35
151.40
151.45
Chromosome 7, position (Mb)

d)

(i)

(ii)

(iii)

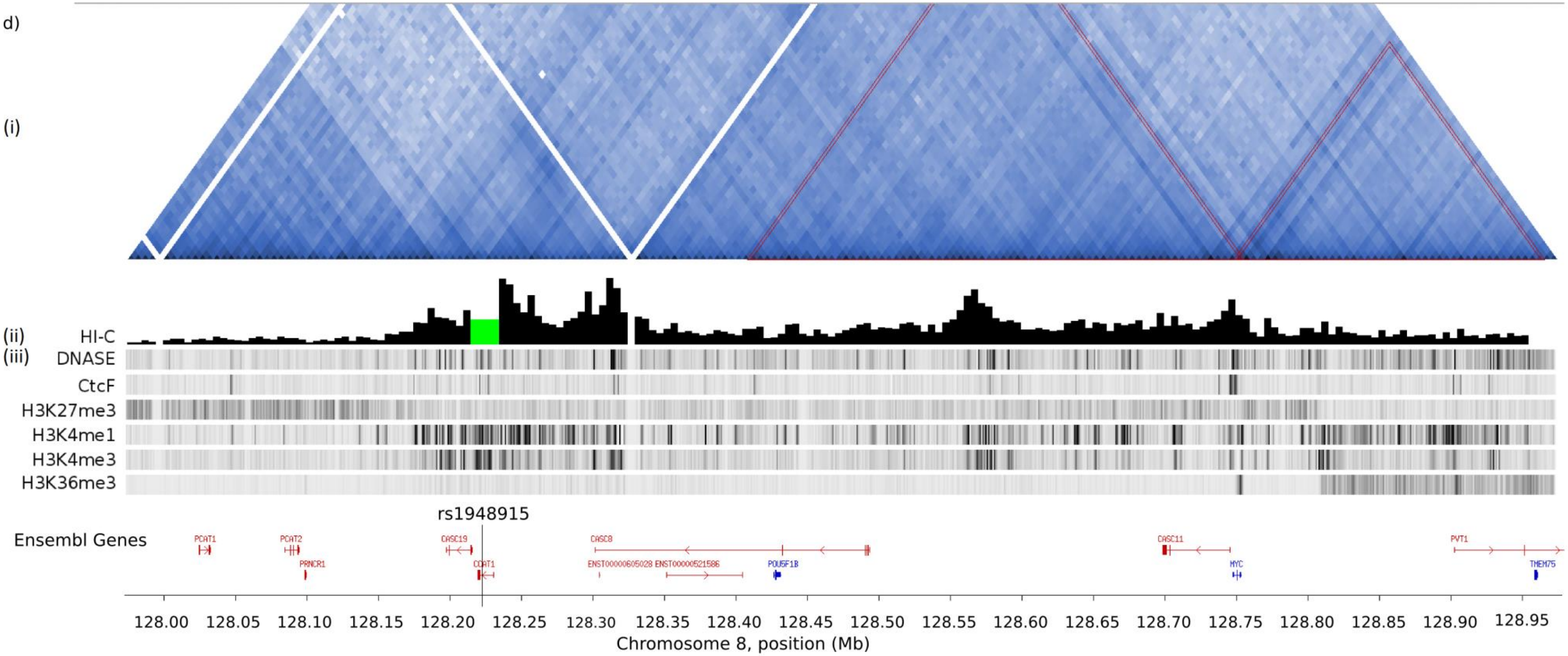

HI-C
DNASE
CtcF
H3K27me3
H3K4me1
H3K4me3
H3K36me3
rs1948915
Ensembl Genes
PCAT1
PCAT2
PRNCR1
CASC19
CCAT1
CASC8
ENST00000605028
ENST00000521586
POU5F1B
CASC11
MYC
PVT1
TMEM75
128.00
128.05
128.10
128.15
128.20
128.25
128.30
128.35
128.40
128.45
128.50
128.55
128.60
128.65
128.70
128.75
128.80
128.85
128.90
128.95
Chromosome 8, position (Mb)

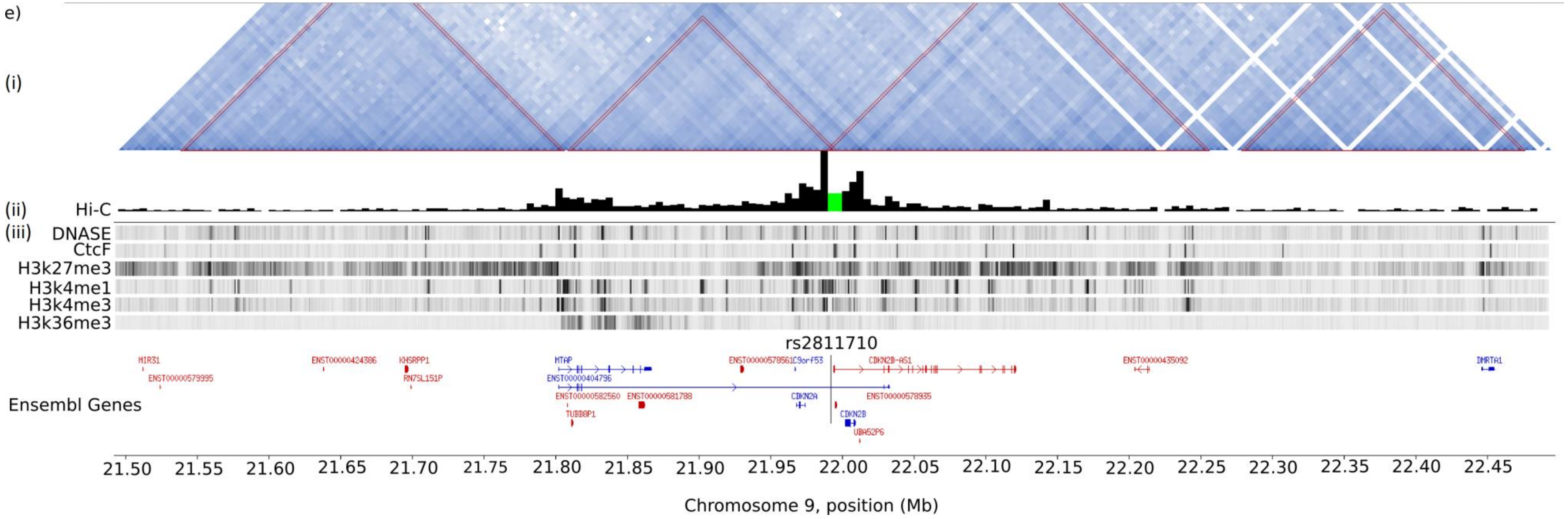
e)
(i)
(ii) Hi-C
(iii) DNASE
CtcF
H3k27me3
H3k4me1
H3k4me3
H3k36me3
rs2811710
Ensembl Genes
21.50 21.55 21.60 21.65 21.70 21.75 21.80 21.85 21.90 21.95 22.00 22.05 22.10 22.15 22.20 22.25 22.30 22.35 22.40 22.45
Chromosome 9, position (Mb)

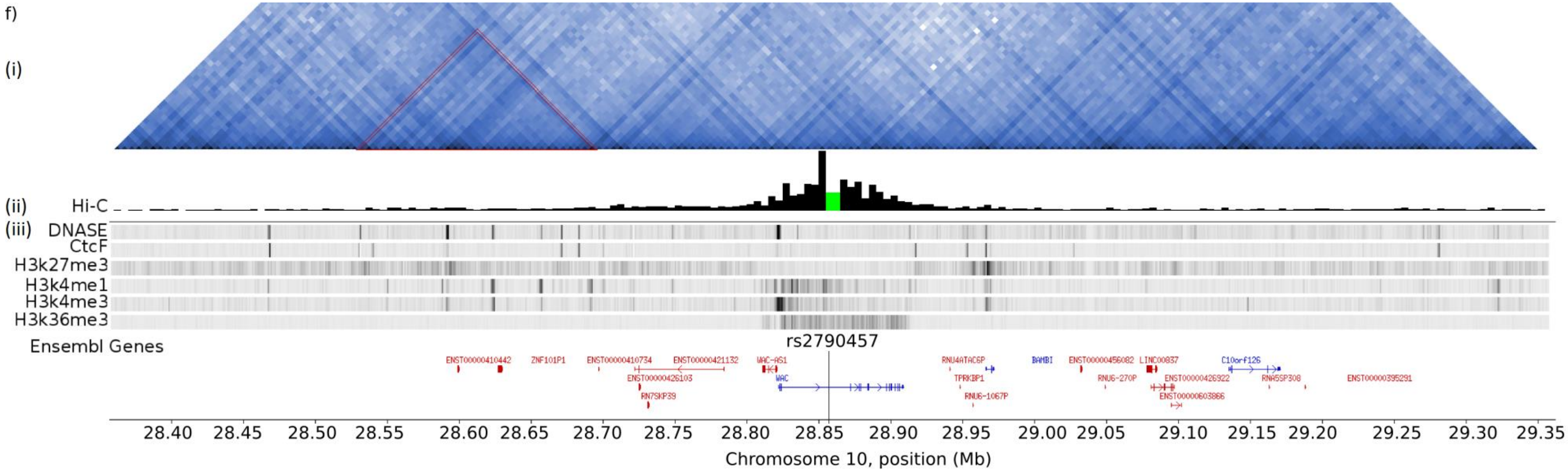
f)
(i)
(ii)
Hi-C
(iii)
DNASE
CtcF
H3k27me3
H3k4me1
H3k4me3
H3k36me3
Ensembl Genes
rs2790457
ENST00000410442
ZNF101P1
ENST00000410734
ENST00000421132
ENST00000426103
RN7SKP39
WAC-AS1
WAC
RNU4ATAC6P
TPRKBP1
RNU6-1067P
BAMBI
ENST00000456082
LINC00837
RNU6-270P
ENST00000426922
ENST00000603866
C10orf126
RNA5SP308
ENST00000395291
28.40
28.45
28.50
28.55
28.60
28.65
28.70
28.75
28.80
28.85
28.90
28.95
29.00
29.05
29.10
29.15
29.20
29.25
29.30
29.35
Chromosome 10, position (Mb)

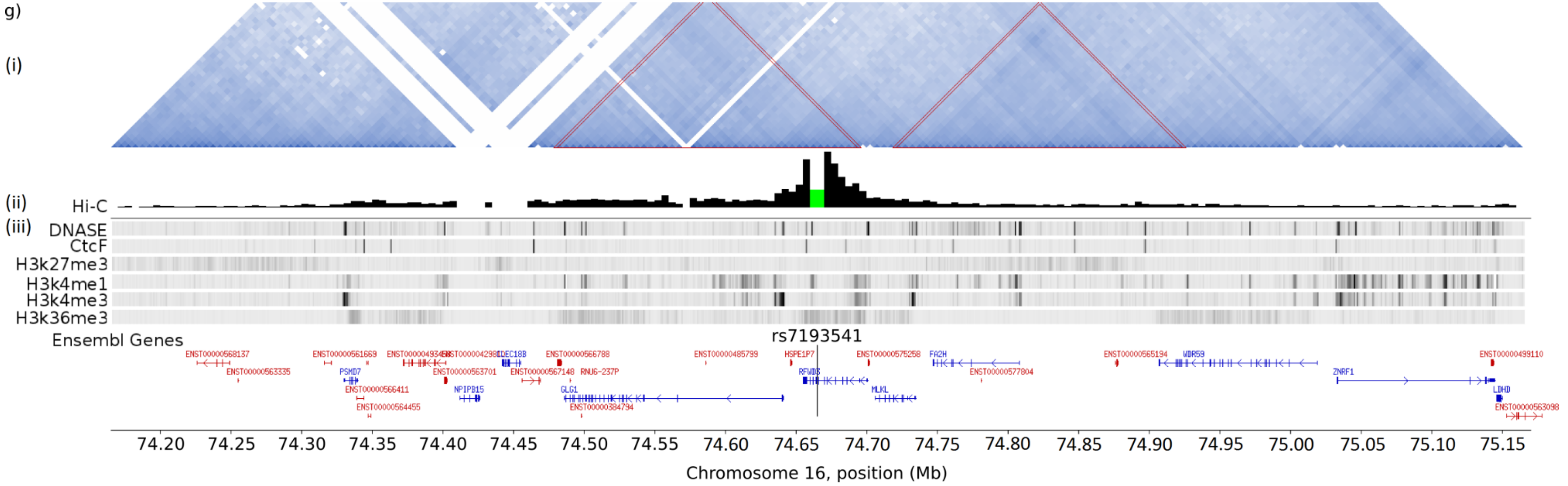

g)
(i)
(ii)
Hi-C
(iii)
DNASE
CtcF
H3k27me3
H3k4me1
H3k4me3
H3k36me3
Ensembl Genes
rs7193541
PSMD7
NPIPB15
GLG1
HSPE1P7
RFWD3
MLKL
FA2H
WDR59
ZNRF1
LDHD
RNU6-237P
74.20 74.25 74.30 74.35 74.40 74.45 74.50 74.55 74.60 74.65 74.70 74.75 74.80 74.85 74.90 74.95 75.00 75.05 75.10 75.15
Chromosome 16, position (Mb)

h)

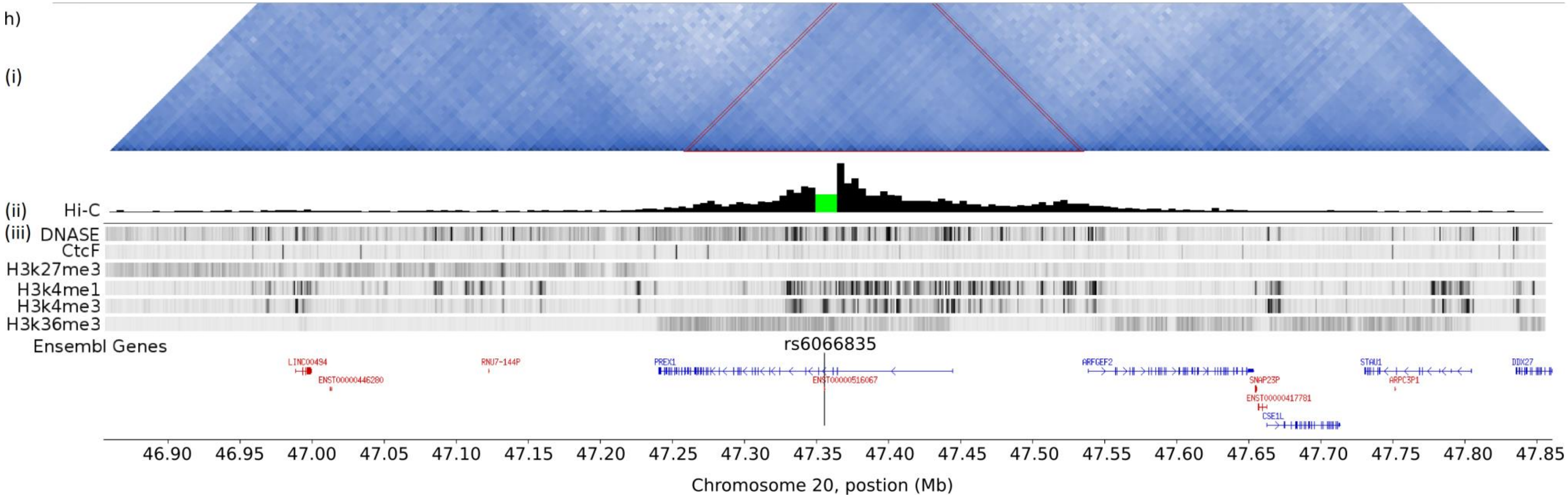

Supplementary Figure 3: Definition of topological domains (TADs) at risk loci and patterns of local chromatin interactions in GM12878[1].
(i) The upper panel shows heat map of chromatin interactions. TADs at each locus (double-red line) were inferred by the Arrowhead method and are superimposed. (ii) Middle panel denotes virtual 4C experiment defined by Hi-C intensity according to viewpoint based on sentinel SNP. The green box denotes a 5Kb (masked) region flanking the sentinel SNP. (iii) Lower panel shows DNase, CTCF, H3K27me3, H3K4me1, H3K4me3 and H3K36me3 tracks for GM12878.

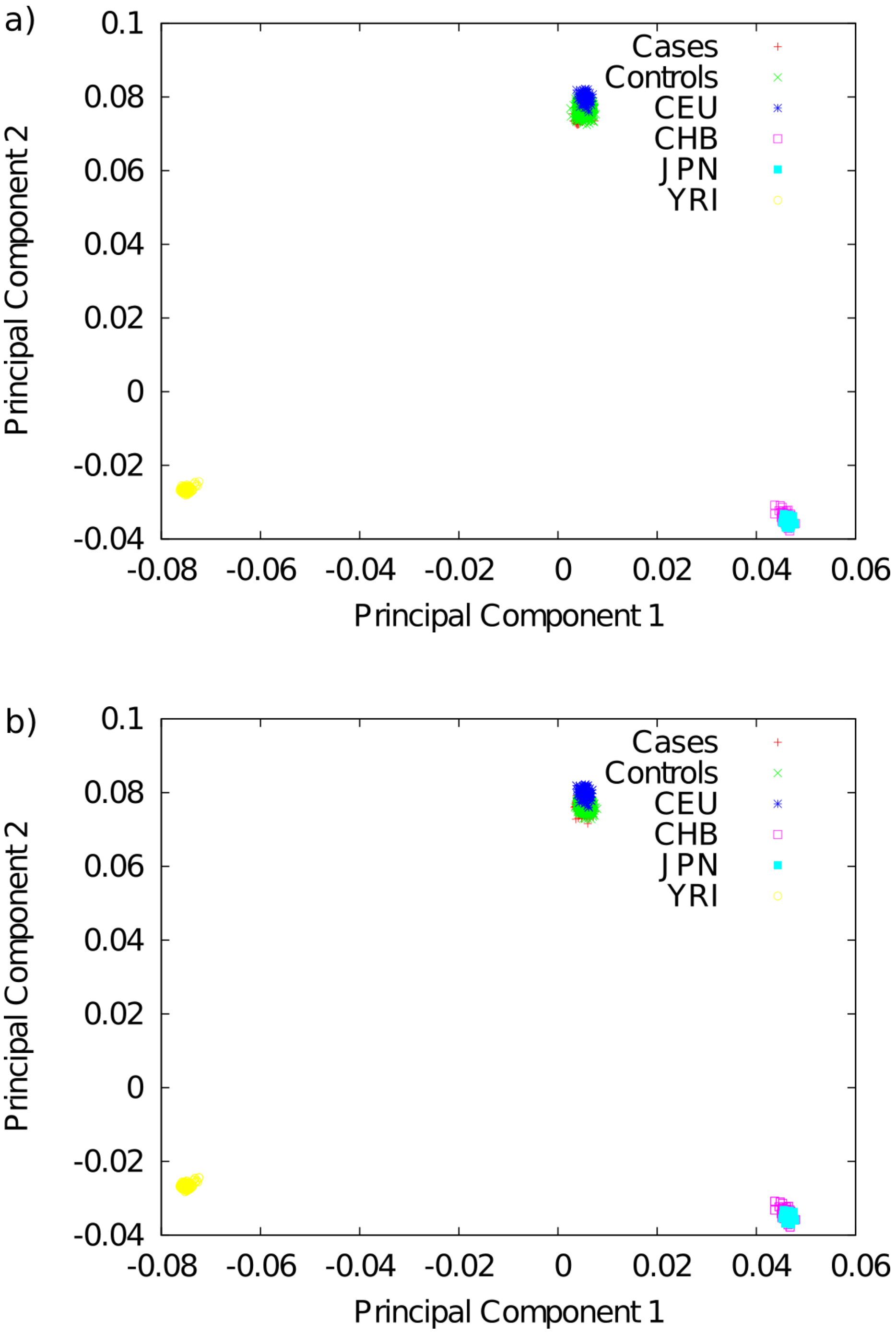
a)
b)
Principal Component 1
Principal Component 2
Cases
Controls
CEU
CHB
JPN
YRI
0.1
0.08
0.06
0.04
0.02
0
-0.02
-0.04
-0.08
-0.06
-0.04
-0.02
0
0.02
0.04
0.06

c)

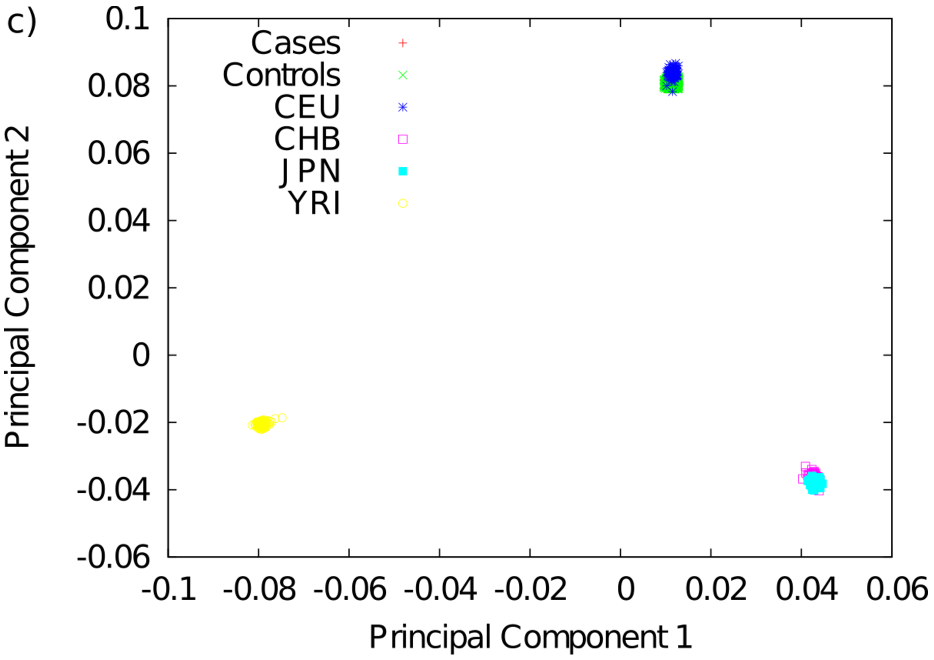

d)

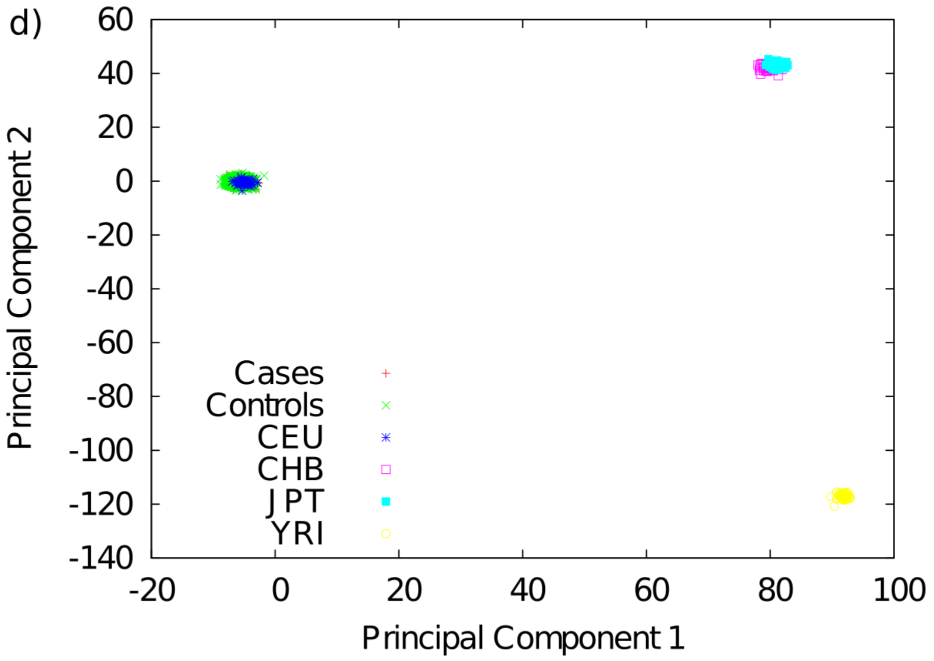

Supplementary Figure 4: Identification of individuals of non-European ancestry in cases and controls. (a) UK-GWAS (Thresholds; 0.0025<PC1<0.0076, 0.0724<PC2<0.0804), (b) German-GWAS (Thresholds; 0.0033<PC1<0.0079, 0.0716<PC2<0.0803), (c) USA-GWAS (Thresholds; 0.0096<PC1<0.0129, 0.0791<PC2<0.0829) and (d) Netherlands-GWAS (Thresholds; -8.8<PC1<-1.7, -3.5<PC2<3.1). The first two principal components of the analysis are plotted. HapMap CEU individuals are plotted in blue; CHB individuals are plotted in indigo; JPN are plotted in cyan; YRI individuals are plotted in yellow. Cases are plotted in red, controls plotted in green, and have been removed if observed here to be of non-European ancestry.

	UK Cases	Controls	Sweden/Norway Cases	Controls	Germany Cases	Controls	Netherlands Cases	Controls	USA Cases	Controls	Iceland Cases	Controls
Pre-QC	2,329	5,199			1,512	2,107	608	2669	1,076	2,234		
Sex discrepancy	10	0			1	0	0	0	0	0		
Call rate fail	1	0			0	0	2	0	0	4		
Heterozygosity rate	NA	NA			NA	NA	7	0	9	2		
Related Individuals	2	2			0	0	0	0	1	0		
Non-European Ancestary	34	0			3	0	44	0	286	369		
Post-QC	2,282	5,197	1,714	10,391	1,508	2,107	555	2669	780	1,857	480	212,164

Supplementary Table 1: Details of the quality control filters applied to each GWAS. Samples were excluded due to call rate (<95% or failed genotyping), ethnicity (principle components analysis or other samples reported to be not of white, European descent), relatedness (any individuals found to be duplicated or related within or between data sets through IBS) or sex discrepancy. Sweden/Norway and Iceland: These studies have been previously reported in their entirety with full QC details[2].

	UK	Sweden/Norway	Germany	Netherlands	USA	Iceland
Pre-QC	409,429		401,405	646,124	296,998	
Call rate fail	997		113	6,523	4	
HWE fail	7		0	18,104	171	
MAF < 0.01	3		1	0	9151	
Post-QC	408,422		401,291	621,497	287,672	
Imputed (filtered)	8,517,071	7,182,761	8,282,831	8,628,799	8,085,846	10,291,845

Supplementary Table 2: Details of the quality control filters applied to each GWAS. Genotyped SNPs with a call rate <95% were excluded as were those with a MAF<0.01 or displaying significant deviation from Hardy-Weinberg equilibrium (*i.e.* $P < 10^{-5}$). Imputed SNPs with information score <0.8 and MAF <0.005 were excluded. Sweden/Norway and Iceland: These studies have been previously reported in their entirety with full QC details[2].

Locus	Published SNP	Strongest signal (current GWAS)	Position (hg19, bp)	Risk Allele	Literature OR (95% CI)	Literature *P* value	Current GWAS OR (95% CI)	Current GWAS *P* value	Reference
2p23.3	rs6746082		25659244	A	1.29 (1.17-1.42)	1.22×10^{-7}	1.20 (1.15-1.26)	2.28×10^{-14}	3
		rs7577599	25613146	T	$r^2 = 0.483$	D' = 0.713	1.24 (1.18-1.30)	1.24×10^{-16}	
3p22.1	rs1052501		41925398	C	1.32 (1.20-1.45)	7.47×10^{-9}	1.25 (1.18-1.31)	8.19×10^{-17}	3
		rs6599192	41992408	G	$r^2 = 0.309$	D' = 0.643	1.26 (1.20-1.33)	8.75×10^{-18}	
7p15.3	rs4487645		21938240	C	1.38 (1.28-1.50)	3.33×10^{-15}	1.24 (1.19-1.30)	5.30×10^{-25}	3
		rs4487645							
3q26.2	rs10936599		169492101	C	1.26 (1.18-1.33)	8.70×10^{-14}	1.20 (1.14-1.25)	9.35×10^{-15}	4
		rs10936600	169514585	A	$r^2 = 1$	D' = 1	1.20 (1.15-1.26)	5.94×10^{-15}	
6p21.3	rs2285803		31107258	T	1.19 (1.13-1.26)	9.67×10^{-11}	1.19 (1.14-1.24)	1.04×10^{-16}	4
		rs3132535	31116526	A	$r^2 = 0.94$	D' = 0.98	1.20 (1.15-1.25)	2.97×10^{-17}	
17p11.2	rs4273077		16849139	G	1.26 (1.16-1.36)	7.67×10^{-9}	1.29 (1.21-1.37)	7.35×10^{-15}	4
		rs34562254	16842991	A	$r^2 = 0.904$	D' = 0.958	1.30 (1.23-1.39)	3.63×10^{-17}	
22q13.1	rs877529		39542292	A	1.23 (1.17-1.29)	7.63×10^{-16}	1.23 (1.19-1.28)	6.07×10^{-26}	4
		rs139402	39546145	C	$r^2 = 1$	D' = 1	1.23 (1.19-1.28)	4.98×10^{-26}	
5q15	rs56219066		95242931	T	1.25 (1.16-1.34)	9.6×10^{-10}	1.16 (1.10-1.22)	8.06×10^{-8}	5
		rs1423269	95255724	A	$r^2 = 0.962$	D' = 0.981	1.17 (1.12-1.22)	1.57×10^{-11}	
22q13	rs138740		35699582	C	1.18 (1.11-1.25)	5.7×10^{-8}	1.11 (1.07-1.16)	2.31×10^{-7}	5
		rs138747	35700488	A	$r^2 = 0.955$	D' = 0.983	1.21 (1.13-1.30)	2.58×10^{-8}	

Supplementary Table 3: Strongest association signals from previously published risk loci discovered in European populations[3-5]. Shown for each region are the published SNP, the most associated variant within a 500kb window in the imputation and the odds ratio and *P* values associated with each, along with the linkage disequilibrium metrics between the SNPs. Meta-analysis for current GWAS was undertaken using the inverse-variance approach under a fixed effects model.

SNP		rs34229995	rs9372120	rs7781265	rs1948915	rs2811710	rs2790457	rs7193541	rs6066835
Locus		6p22.3	6q21	7q36.1	8q24.21	9p21.3	10p12.1	16q23.1	20q13.13
Position (bp, hg19)		15,244,018	106,667,535	150,950,940	128,222,421	21,991,923	28,856,819	74,664,743	47,355,009
Allele A		C	T	C	T	A	A	C	T
Allele B (Risk Allele)		G	G	T	C	G	G	T	C
UK	Cases RAF	0.024	0.236	0.128	0.332	0.666	0.751	0.628	0.100
	Controls RAF	0.019	0.211	0.117	0.319	0.634	0.733	0.584	0.083
	OR	1.28	1.16	1.11	1.06	1.16	1.10	1.20	1.25
	P value	0.0525	6.72×10^{-4}	0.0578	0.109	1.24×10^{-4}	0.0149	5.97×10^{-7}	3.83×10^{-4}
Sweden/ Norway	Cases RAF	0.045	0.243		0.386	0.681	0.750	0.598	0.097
	Controls RAF	0.033	0.217		0.363	0.660	0.735	0.572	0.075
	OR	1.44	1.22		1.12	1.14	1.11	1.07	1.38
	P value	4.37×10^{-4}	9.96×10^{-6}	Info<0.8	2.97×10^{-3}	1.38×10^{-3}	0.0160	0.0612	6.20×10^{-6}
Germany	Cases RAF	0.039	0.226	0.144	0.357	0.678	0.757	0.596	0.095
	Controls RAF	0.028	0.207	0.118	0.331	0.654	0.728	0.590	0.077
	OR	1.49	1.18	1.33	1.22	1.15	1.15	1.02	1.24
	P value	9.03×10^{-3}	0.0124	3.22×10^{-4}	3.13×10^{-4}	0.0119	0.0224	0.645	0.0240
Iceland	Cases RAF								
	Controls RAF								
	OR	1.32	1.19	1.14	1.21	1.06	1.19	1.14	1.08
	P value	0.0110	0.0501	0.190	0.0153	0.440	0.0402	0.0907	0.535
USA	Cases RAF	0.036	0.239	0.159	0.362	0.669	0.761	0.604	0.105
	Controls RAF	0.023	0.202	0.120	0.313	0.637	0.732	0.584	0.082
	OR	1.61	1.25	1.45	1.25	1.16	1.17	1.09	1.33
	P value	0.0110	2.46×10^{-3}	6.56×10^{-5}	5.35×10^{-4}	0.0258	0.0232	0.166	7.11×10^{-3}
Netherlands	Cases RAF	0.031	0.239	0.137	0.363	0.682	0.749	0.616	0.081
	Controls RAF	0.024	0.204	0.124	0.329	0.658	0.742	0.570	0.085
	OR	1.32	1.23	1.12	1.17	1.12	1.04	1.21	0.95
	P value	0.192	9.45×10^{-3}	0.240	0.0258	0.113	0.620	4.36×10^{-3}	0.687
Meta	OR	1.40	1.20	1.20	1.14	1.14	1.12	1.12	1.24
	(95% CI)	(1.25-1.58)	(1.14-1.26)	(1.12-1.29)	(1.10-1.19)	(1.09-1.19)	(1.07-1.17)	(1.08-1.17)	(1.16-1.33)
	P value	1.76×10^{-8}	8.72×10^{-14}	1.82×10^{-7}	3.14×10^{-10}	6.50×10^{-10}	8.44×10^{-7}	1.14×10^{-8}	1.16×10^{-9}

Supplementary Table 4: Summary statistics for novel variants showing an association with multiple myeloma risk in the GWAS meta-analysis at *P* < 1.0 × 10^{-6}. Showing SNPs which were genome-wide significant after replication. Odds ratios derived with respect to the risk allele. Cases RAF, risk allele frequency in discovery cases. Controls RAF, risk allele frequency in discovery controls. Shown are discovery association *P* values for individual studies (logistic regression) and from meta-analysis of the six GWAS datasets (inverse-variance approach under a fixed effects model).

SNP		rs78311596	rs72665486	rs1034447	rs17507636	rs28571765	rs76601148	rs8058578	rs6071887
Locus		1p13.2	4q22.1	6q27	7q22.3	8q11.23	13q13.3	16p11.2	20q12
Position (bp, hg19)		113,300,945	91,913,730	164,613,457	106,291,118	55,449,281	36,746,056	30,726,248	38,621,456
Allele A		A	T	C	T	C	A	C	G
Allele B (Risk Allele)		T	G	T	C	T	G	T	A
UK	Cases RAF	0.029	0.882	0.163	0.760	0.808	0.049	0.280	0.258
	Controls RAF	0.022	0.874	0.148	0.745	0.794	0.041	0.255	0.238
	OR	1.34	1.07	1.13	1.08	1.09	1.07	1.14	1.11
	P value	0.0138	0.180	0.0148	0.0476	0.0532	0.0152	1.34×10^{-3}	8.42×10^{-3}
Sweden/ Norway	Cases RAF		0.907	0.158	0.778	0.797	0.051	0.283	0.225
	Controls RAF		0.886	0.127	0.765	0.773	0.038	0.261	0.208
	OR		1.25	1.27	1.09	1.13	1.39	1.11	1.11
	P value	MAF<0.005	2.14×10^{-4}	1.40×10^{-5}	0.0436	8.80×10^{-3}	6.57×10^{-4}	0.0126	0.0199
Germany	Cases RAF	0.020	0.907	0.142	0.745	0.825	0.039	0.281	0.220
	Controls RAF	0.016	0.883	0.138	0.714	0.812	0.033	0.261	0.213
	OR	1.449	1.36112	1.0950	1.13882	1.09694	1.4424	1.0979	1.06395
	P value	0.0733	2.9×10^{-4}	0.238	0.0281	0.175	0.0111	0.115	0.331
Iceland	Cases RAF								
	Controls RAF								
	OR	1.71	1.21	1.13	1.06	1.30	1.07	1.19	1.12
	P value	1.47×10^{-3}	0.0970	0.226	0.491	3.75×10^{-3}	0.692	0.0350	0.213
USA	Cases RAF	0.027	0.887	0.144	0.785	0.826	0.051	0.273	0.267
	Controls RAF	0.017	0.884	0.136	0.737	0.789	0.041	0.254	0.231
	OR	1.73	1.02	1.06	1.30	1.26	1.28	1.10	1.22
	P value	0.0132	0.811	0.489	2.03×10^{-4}	2.31×10^{-3}	0.0963	0.160	4.90×10^{-3}
Netherlands	Cases RAF	0.026	0.896	0.153	0.764	0.825	0.059	0.259	0.251
	Controls RAF	0.021	0.878	0.141	0.735	0.803	0.043	0.245	0.221
	OR	1.26	1.19	1.10	1.16	1.16	1.45	1.07	1.17
	P value	0.321	0.0870	0.300	0.0440	0.0776	0.0206	0.259	0.0380
Meta	OR	1.46	1.17	1.15	1.12	1.14	1.31	1.12	1.12
	(95% CI)	(1.25-1.69)	(1.10-1.24)	(1.09-1.22)	(1.07-1.17)	(1.08-1.19)	(1.19-1.45)	(1.07-1.17)	(1.07-1.18)
	P value	9.40×10^{-7}	4.81×10^{-7}	8.21×10^{-7}	5.54×10^{-7}	1.30×10^{-7}	5.90×10^{-8}	5.57×10^{-7}	7.08×10^{-7}

Supplementary Table 5: Summary statistics for novel variants showing an association with multiple myeloma risk in the GWAS meta-analysis at *P* < 1.0 × 10^{-6}. Showing SNPs which were not genome-wide significant after replication. Odds ratios derived with respect to the risk allele. Cases RAF, risk allele frequency in discovery cases. Controls RAF, risk allele frequency in discovery controls. Shown are discovery association *P* values for individual studies (logistic regression) and from meta-analysis of the six GWAS datasets (inverse-variance approach under a fixed effects model).

SNP		rs34229995	rs9372120	rs7781265	rs1948915	rs2811710	rs2790457	rs7193541	rs6066835
UK Cases	AA	164/164	105/105	126/127	77/77	61/62	97/97	Genotyped	130/130
	Aa	7/7	57/59	39/40	68/68	71/73	65/65		41/41
	aa	2/2	7/7	3/3	13/14	23/23	12/12		2/2
	r^2	1.00	1.0	0.97	0.99	0.98	1.00		1.00
UK Controls	AA	146/146	110/110	123/123	75/77	69/70	85/87		142/142
	Aa	16/16	52/52	30/32	64/64	62/63	63/64		21/21
	aa	1/1	1/1	2/2	12/13	15/16	12/12		NA
	r^2	1.00	1.00	0.97	0.98	0.98	0.99		1.00
Germany Cases	AA	314/314	193/193	250/254	135/136	146/153	184/185		279/279
	Aa	20/20	124/125	68/70	142/148	134/138	124/124		53/55
	aa	1/1	12/12	6/6	36/38	26/27	26/26		3/3
	r^2	1.00	0.99	0.95	0.97	0.94	1.00		0.98
USA Cases	AA	692/692	428/431	494/506	307/308	308/314	420/421		591/591
	Aa	50/50	268/269	149/159	320/322	296/299	276/278		143/143
	aa	2/2	41/41	13/14	103/104	72/74	38/38		6/6
	r^2	1.00	0.98	0.86	0.99	0.96	0.99		1.0

Supplementary Table 6: Concordance between directly sequenced and imputed genotype. Showing SNPs which were genome-wide significant after replication. AA, major homozygote; AB, heterozygote; BB, minor homozygote. r^2 indicates Pearson product-moment correlation coefficient between imputed and sequenced genotype.

Study	Genotype	rs78311596	rs72665486	rs1034447	rs17507636	rs28571765	rs76601148	rs8058578	rs6071887
UK Cases	AA	166/166	127/127	120/120	Genotyped	108/108	151/151	Genotyped	97/97
	Aa	8/9	35/35	48/48		61/62	22/22		59/59
	aa	N/A	3/3	6/6		4/4	2/2		15/15
	r^2	0.94	1.00	1.00		0.99	1.00		1.00
UK Controls	AA	142/142	124/125	126/126		101/101	105/105		85/85
	Aa	17/18	37/37	35/35		52/52	56/56		70/70
	aa	2/2	1/1	1/1		8/8	2/2		3/3
	r^2	0.98	1.00	1.00		1.00	1.00		1.00
Germany Cases	AA	318/318	138/138	246/247		228/229	317/318		189/202
	Aa	9/9	32/32	87/87		91/91	19/20		109/109
	aa	NA	NA	5/5		9/9	NA		14/14
	r^2	1.00	1.00	0.99		0.99	0.97		0.91
USA Cases	AA	704/705	587/587	539/540		512/512	672/672		396/396
	Aa	28/34	154/154	189/191		193/193	71/71		93/293
	aa	1/1	8/9	10/10		29/29	2/2		49/49
	r^2	0.80	0.99	0.98		1.0	1.0		1.0

Supplementary Table 7: Concordance between directly sequenced and imputed genotype. Showing SNPs which were not genome-wide significant after replication. AA, major homozygote; AB, heterozygote; BB, minor homozygote. r^2 indicates Pearson product-moment correlation coefficient between imputed and sequenced genotype.

SNP		rs34229995	rs9372120	rs7781265	rs1948915	rs2811710	rs2790457	rs7193541	rs6066835
Locus		6p22.3	6q21	7q36.1	8q24.21	9p21.3	10p12.1	16q23.1	20q13.13
Position (bp, hg19)		15,244,018	106,667,535	150,950,940	128,222,421	21,991,923	28,856,819	74,664,743	47,355,009
Allele A		C	T	C	T	A	A	C	T
Allele B (Risk Allele)		G	G	T	C	G	G	T	C
UK	Cases RAF	0.022	0.234	0.136	0.332	0.666	0.752	0.63	0.101
	Controls RAF	0.020	0.210	0.113	0.309	0.634	0.733	0.58	0.083
	OR	1.14	1.15	1.23	1.11	1.12	1.07	1.11	1.37
	P value	0.576	0.091	0.043	0.151	0.106	0.423	0.147	0.008
Sweden/Norway	Cases RAF	0.010	0.210	0.143	0.404	0.710	0.758	0.618	0.070
	Controls RAF	0.020	0.201	0.125	0.366	0.666	0.729	0.583	0.071
	OR	0.59	1.052	1.171	1.17	1.23	1.17	1.156	0.99
	P value	0.128	0.677	0.267	0.106	0.053	0.164	0.142	0.974
Germany	Cases RAF	0.024	0.211	0.134	0.350	0.669	0.759	0.614	0.102
	Controls RAF	0.015	0.197	0.127	0.330	0.630	0.727	0.565	0.805
	OR	1.60	1.09	1.06	1.10	1.19	1.190	1.22	1.29
	P value	0.03	0.26	0.51	0.16	0.009	0.02	0.001	0.015
Denmark	Cases RAF	0.012	0.229	0.147	0.365	0.698	0.751	0.616	0.121
	Controls RAF	0.012	0.199	0.121	0.366	0.651	0.733	0.590	0.074
	OR	1.062	1.19	1.25	1.00	1.24	1.10	1.114	1.71
	P value	0.870	0.081	0.069	0.960	0.019	0.349	0.228	1×10^{-4}
Discovery phase + replication	OR	1.37	1.18	1.19	1.13	1.15	1.12	1.13	1.26
	P value	1.31×10^{-8}	9.09×10^{-15}	9.71×10^{-9}	4.20×10^{-11}	1.72×10^{-13}	1.77×10^{-8}	5.00×10^{-12}	1.36×10^{-13}

Supplementary Table 8: Replication of top association signals. Showing SNPs which were genome-wide significant after replication. Cases RAF, risk allele frequency of replication cases; Control RAF, risk allele frequency of replication controls. *P* values are shown for each replication series (logistic regression) and for the meta-analysis of the 6 discovery datasets and the four replication series combined (inverse-variance approach under a fixed effects model).

SNP		rs72665486	rs1034447	rs17507636	rs28571765	rs76601148	rs8058578	rs6071887
Locus		4q22.1	6q27	7q22.3	8q11.23	13q13.3	16p11.2	20q12
Position (bp, hg19)		91,913,730	164,613,457	106,291,118	55,449,281	36,746,056	30,726,248	38,621,456
Allele A		T	C	T	C	A	C	G
Allele B (Risk Allele)		G	T	C	T	G	T	A
UK	Cases RAF	0.882	0.163	0.760	0.808	0.049	0.280	0.258
	Controls RAF	0.874	0.148	0.745	0.794	0.041	0.255	0.238
	OR	1.07	1.02	1.09	1.15	1.07	1.15	1.10
	P value	0.440	0.812	0.312	0.098	0.694	0.064	0.214
Sweden/Norway	Cases RAF	0.871	0.155	0.784	0.790	0.040	Genotyping failed	0.188
	Controls RAF	0.879	0.139	0.764	0.785	0.039		0.212
	OR	0.93	1.14	1.27	1.03	1.04		0.86
	P value	0.639	0.400	0.308	0.784	0.882		0.236
Germany	Cases RAF	0.893	0.138	0.760	0.810	0.366	0.296	0.232
	Controls RAF	0.884	0.131	0.735	0.807	0.410	0.304	0.216
	OR	1.10	1.06	1.15	1.02	0.89	0.96	1.10
	P value	0.34	0.51	0.06	0.81	0.47	0.59	0.23
Denmark	Cases RAF	0.884	0.168	0.734	0.766	0.033	Genotyping failed	0.223
	Controls RAF	0.889	0.146	0.751	0.80	0.039		0.215
	OR	0.95	1.18	0.93	0.79	0.85		1.05
	P value	0.709	0.171	0.434	0.0182	0.455		0.654
Discovery phase + replication	OR	1.14	1.13	1.11	1.10	1.23	1.10	1.11
	P value	2.15×10^{-6}	4.81×10^{-7}	1.58×10^{-7}	1.58×10^{-6}	4.46×10^{-6}	9.68×10^{-7}	6.47×10^{-7}

Supplementary Table 9: Replication of top association signals. Showing SNPs which were not genome-wide significant after replication. Cases RAF, risk allele frequency of replication cases; Control RAF, risk allele frequency of replication controls. *P* values are shown for each replication series (logistic regression) and for the meta-analysis of the 6 discovery datasets and the four replication series combined (inverse-variance approach under a fixed effects model).

Cancer	SNP	Reference	Previously Reported		Current MM GWAS		LD with rs1948915	
			Risk Allele	*P* value	Risk Allele	*P* value	r^2	D'
Hodgkin's Lymphoma	rs2019960	6	C	7×10^{-8}	C	0.024	1.9×10^{-5}	-0.012
Colorectal Cancer	rs6983267	7	G	5×10^{-14}	T	0.17	8.7×10^{-4}	-0.046
Prostate Cancer	rs1447295	8	A	6×10^{-18}	C	0.55	1.6×10^{-4}	0.055
Prostate Cancer	rs12682344	8	G	5×10^{-12}	G	0.48	5.2×10^{-3}	-0.57
Prostate Cancer (early onset)	rs10505477	9	A	9×10^{-9}	G	0.11	1.1×10^{-3}	-0.051
Colorectal Cancer	rs10505477	10	T	8×10^{-13}	G	0.11	1.1×10^{-3}	-0.051
Breast Cancer (early onset)	rs2392780	11		1×10^{-8}	G	0.86	1.0×10^{-3}	0.039
Renal cell carcinoma	rs6470589	12	G	5×10^{-11}	G	0.82		
Bladder Cancer	rs9642880	13	T	4×10^{-38}	T	0.17	3.9×10^{-5}	8.6×10^{-3}
Chronic lymphocytic leukemia	rs2466035	14	C	2×10^{-8}	C	3.6×10^{-3}	0.15	0.39
Breast Cancer	rs13281615	15	G	1×10^{-17}	G	0.076	6.8×10^{-4}	-0.046
Breast Cancer	rs11780156	15	T	3×10^{-11}	T	0.22	2.8×10^{-5}	0.0076
Ovarian Cancer	rs10088218	16		1×10^{-17}	A	0.86	1.0×10^{-3}	-0.13
Prostate Cancer	rs6983561	17	C	4×10^{-13}	C	0.47	4.9×10^{-3}	-0.55
Prostate Cancer	rs13254738	18	C	4×10^{-10}	A	0.43	0.030	-0.17
Prostate Cancer	rs4242384	19	C	3×10^{-16}	A	0.57	2.1×10^{-4}	0.063
Prostate Cancer	rs1016343	19	T	4×10^{-10}	T	0.63	0.015	0.16
Glioma	rs4295627	20	G	5×10^{-21}	G	5.4×10^{-3}	6.3×10^{-4}	0.0047
Breast Cancer	rs1562430	21	A	3×10^{-11}	C	0.65	8.9×10^{-4}	0.037
Prostate Cancer	rs1456315	22	A	2×10^{-29}	C	0.26	0.028	-0.17
Prostate Cancer	rs7837688	22	T	1×10^{-25}	G	0.68	3.7×10^{-4}	0.083
Prostate Cancer	rs16902094	23	G	6×10^{-15}	G	0.74	1.9×10^{-4}	-0.049
Prostate Cancer	rs16901979	23	A	3×10^{-14}	A	0.50	5.2×10^{-3}	-0.57
Prostate Cancer	rs445114	23	T	5×10^{-10}	C	0.13	1.6×10^{-3}	-0.076
Glioma	rs891835	24	G	8×10^{-11}	G	0.039	2.1×10^{-5}	5.8×10^{-3}
Colorectal Cancer	rs7014346	25	A	9×10^{-26}	G	7.9×10^{-4}	2.2×10^{-4}	0.028
Prostate Cancer	rs6983267	26	G	7×10^{-12}	T	0.17	8.7×10^{-4}	-0.046
Glioma	rs55705857	27	A	2.24×10^{-38}	A	9.5×10^{-4}	1.2×10^{-5}	-0.017

Supplementary Table 10: Previously reported 8q24.21 cancer associated SNPs and their LD with rs1948915[6-27].

Cancer	SNP	Reference	Previously Reported Risk Allele	Previously Reported P value	Current MM GWAS Risk Allele	Current MM GWAS P value	LD with rs2811710 r^2	LD with rs2811710 D'
Basal cell carcinoma	rs2151280	28	G	3×10^{-10}	A	0.0127	4.14×10^{-4}	-0.0280
ALL	rs3731249	29	T	9.4×10^{-23}	T	0.957	0.0385	0.920
CLL	rs1679013	30	C	1×10^{-8}	C	6.54×10^{-3}	4.27×10^{-3}	0.0791
Glioma	rs4977756	31	G	1×10^{-8}	A	0.368	0.0513	0.360
Melanoma	rs7023329	32	A	7×10^{-9}	G	0.0822	1.06×10^{-4}	-0.0131
Glioma	rs2157719	20	C	5×10^{-16}	T	0.399	0.0485	0.328
Glioma (High Grade)	rs1412829	33	C	2×10^{-10}	C	0.285	0.0434	-0.317

Supplementary Table 11: Previously reported 9p21.3 cancer associated SNPs and their LD with rs2811710[20,28-33].

		rs34229995	rs9372120	rs7781265	rs1948915	rs2811710	rs2790457	rs7193541	rs6066835
UK	Beta	0.44	0.64	-0.093	-0.43	-0.20	-0.36	-0.022	-0.075
	P value	0.67	0.076	0.84	0.19	0.56	0.31	0.94	0.88
Sweden/	Beta	1.88	1.31	NA	0.33	-0.054	0.60	-0.30	1.82
Norway	*P* value	0.070	9.5×10^{-3}	NA	0.47	0.91	0.23	0.51	0.01
Germany	Beta	0.068	0.15	-0.41	0.34	0.40	0.24	0.22	0.14
	P value	0.94	0.73	0.43	0.35	0.32	0.57	0.56	0.82
USA	Beta	0.93	-1.18	0.023	0.28	0.017	-0.51	0.55	-0.71
	P value	0.45	0.032	0.97	0.56	0.97	0.36	0.25	0.35
Netherlands	Beta	-0.96	-0.11	-0.12	-0.046	-0.45	-0.33	-0.47	-0.21
	P value	0.16	0.67	0.95	0.84	0.049	0.16	0.025	0.57

Supplementary Table 12: Relationship between SNP genotype and age at diagnosis. Analysis based on Beta values calculated from linear regression on the discovery phase data sets from UK (cases=2282, controls=5197), Sweden/Norway (cases=1714, controls=10391), Germany (cases=1508, controls=2107), USA (cases=780, controls=1857) and Netherlands (cases=555, controls=2669).

		rs34229995	rs9372120	rs7781265	rs1948915	rs2811710	rs2790457	rs7193541	rs6066835
UK	Beta	-0.052	-0.013	-0.11	0.036	-0.028	0.12	0.021	0.069
	P value	0.79	0.85	0.22	0.58	0.67	0.081	0.74	0.49
Sweden/	Beta	0.26	-0.020	NA	-0.083	-0.042	-0.12	-0.11	0.11
Norway	*P* value	0.12	0.81	NA	0.25	0.58	0.12	0.13	0.34
Germany	Beta	-0.078	-0.11	0.021	0.049	-0.028	-0.06	-0.012	-0.24
	P value	0.69	0.22	0.84	0.53	0.74	0.45	0.88	0.06
USA	Beta	-0.43	0.16	-0.061	0.019	-0.13	-0.040	-0.046	-0.60
	P value	0.15	0.20	0.69	0.86	0.24	0.75	0.67	1.7×10^{-3}
Netherlands	Beta	-0.11	-0.13	0.077	-0.11	0.017	-0.012	0.028	-0.068
	P value	0.95	0.88	0.46	0.99	0.86	0.90	0.78	0.55

Supplementary Table 13: Relationship between SNP genotype and sex of multiple myeloma cases. Analysis based on Beta values calculated from logistic regression on the discovery phase data sets from UK (cases=2282, controls=5197), Sweden/Norway (cases=1714, controls=10391), Germany (cases=1508, controls=2107), USA (cases=780, controls=1857) and Netherlands (cases=555, controls=2669).

			rs34229995	rs9372120	rs7781265	rs1948915	rs2811710	rs2790457	rs7193541	rs6066835
Hyperdiploid	UK	Beta	0.0285	-0.111	-6.14×10^{-3}	-0.0441	0.0191	-0.142	-0.0374	0.369
	(702)	*P* value	0.892	0.140	0.950	0.522	0.783	0.0555	0.569	5.74×10^{-4}
	Germany (661)	Beta	-0.152	-0.0373	7.33×10^{-3}	0.0802	-1.37×10^{-3}	-0.0318	-0.0804	0.0649
		P value	0.435	0.677	0.944	0.303	0.987	0.713	0.281	0.600
	Meta	Beta	-0.0686	-0.0805	1.17×10^{-4}	0.0104	0.0105	-0.0953	-0.0562	0.239
		P value	0.630	0.162	0.999	0.840	0.843	0.0901	0.255	3.20×10^{-3}
Translocation	UK	Beta	0.0181	0.155	-0.230	0.0758	0.116	-0.137	0.0190	-0.0618
	(571)	*P* value	0.936	0.0517	0.0272	0.302	0.118	0.0818	0.786	0.588
	Germany (768)	Beta	0.187	-8×10^{-4}	-0.0232	-0.0127	-6.96×10^{-3}	-0.0771	-0.0657	-0.151
		P value	0.331	0.992	0.824	0.870	0.931	0.370	0.375	0.219
	Meta	Beta	0.116	0.0857	-0.127	0.0338	0.0596	-0.110	-0.0210	-0.103
		P value	0.429	0.150	0.0854	0.526	0.274	0.0589	0.680	0.217
t(11;14)	UK	Beta	0.0335	0.186	-0.380	0.0845	0.119	-0.322	0.0191	0.171
	(231)	*P* value	0.918	0.104	0.0110	0.423	0.261	4.37×10^{-3}	0.850	0.296
	Germany	Beta	-0.232	0.0900	-0.0818	-0.195	-0.0151	0.0881	-0.0224	-0.130
	(277)	*P* value	0.350	0.434	0.543	0.0510	0.885	0.427	0.815	0.409
	Meta	Beta	-0.134	0.138	-0.215	-0.0628	0.0509	-0.113	-2.69×10^{-3}	0.0151
		P value	0.497	0.0886	0.0311	0.387	0.494	0.153	0.969	0.894
t(4;14)	UK	Beta	-0.392	0.0396	-0.0224	0.154	0.0172	0.176	0.0733	-0.351
	(170)	*P* value	0.292	0.764	0.897	0.204	0.887	0.177	0.526	0.0622
	Germany	Beta	0.521	-0.110	0.206	0.0281	-0.170	-0.122	-0.135	-0.129
	(142)	*P* value	0.112	0.472	0.245	0.831	0.219	0.407	0.289	0.541
	Meta	Beta	0.121	-0.0243	0.0887	0.0963	-0.0644	0.0451	-0.0210	-0.252
		P value	0.622	0.808	0.474	0.281	0.480	0.644	0.806	0.0724
t(14;16)	UK	Beta	1.67	0.309	-0.210	-0.139	-0.189	-0.342	0.0767	-0.896
	(24)	*P* value	0.0778	0.361	0.635	0.657	0.541	0.305	0.798	0.0637
	Germany	Beta	0.384	-0.182	0.205	0.0490	0.235	-0.283	0.0260	-0.487
	(29)	*P* value	0.586	0.587	0.595	0.863	0.420	0.367	0.923	0.284
	Meta	Beta	0.843	0.0659	0.0263	-0.0362	-0.0356	0.311	-0.0486	-0.680
		P value	0.136	0.784	0.928	0.863	0.867	0.174	0.809	0.0403

Supplementary Table 14: Relationship between SNP genotype and multiple myeloma subtype. Case-only analysis; Beta values obtained from logistic regression. FISH and ploidy classification of UK and German samples were determined as previously described[34-36]. Number of samples in each cohort indicated in brackets.

SNP		rs34229995	rs9372120	rs7781265	rs1948915	rs2811710	rs2790457	rs7193541	rs6066835
Locus		6p22.3	6q21	7q36.1	8q24.21	9p21.3	10p12.1	16q23.1	20q13.13
Position (bp, hg19)		15,244,018	106,667,535	150,950,940	128,222,421	21,991,923	28,856,819	74,664,743	47,355,009
Allele A		C	T	C	T	A	A	C	T
Allele B (Risk Allele)		G	G	T	C	G	G	T	C
Nearest Gene		*JARID2*	*ATG5*	*SMARCD3*	*CCAT1*	*CDKN2A*	*WAC*	*RFWD3*	*PREX1*
UK-MyIX	HR	1.48	0.82	0.83	0.98	1.07	0.91	0.94	0.97
	(95%CI)	(1.03-2.12)	(0.71-0.95)	(0.68-1.01)	(0.86-1.13)	(0.94-1.22)	(0.79-1.05)	(0.83-1.07)	(0.79-1.19)
	P value	0.035	0.008	0.06	0.827	0.31	0.208	0.344	0.775
UK-MyXI	HR	0.81	1.19	1.12	1.05	0.91	0.92	1.05	0.88
	(95%CI)	(0.40-1.64	(0.96-1.47)	(0.84-1.49)	(0.85-1.29)	(0.73-1.13)	(0.73-1.16)	(0.87-1.29)	(0.63-1.25)
	P value	0.555	0.117	0.423	0.679	0.409	0.484	0.61	0.484
GER-GMMG	HR	0.89	1.02	1.19	1.04	0.77	0.88	1.01	1.09
	(95%CI)	(0.49-1.63)	(0.76-1.35)	(0.89-1.60)	(0.83-1.31)	(0.59-1.01	(0.67-1.17)	(0.78-1.28)	(0.77-1.54)
	P value	0.710	0.918	0.243	0.731	0.06	0.395	0.943	0.634
US-UAMS	HR	1.01	0.96	1.16	0.96	1.07	0.94	1.06	1.11
	(95%CI)	(0.68-1.51)	(0.80-1.5)	(0.93-1.45)	(0.82-1.12)	(0.87-1.22)	(0.78-1.14)	(0.91-1.24)	(0.87-1.42)
	P value	0.958	0.631	0.174	0.591	0.650	0.552	0.430	0.410
Combined	HR (95%CI)	1.13 (0.90-1.43)	0.94 (0.86-1.04)	1.02 (0.91-1.15)	0.99 (0.91-1.08)	1.01 (0.92-1.10)	0.92 (0.83-1.01)	1.00 (0.92-1.09)	1.01 (0.89-1.15)
	I^2	22	64	55	0	50	0	50	0
	P value	0.295	0.206	0.698	0.894	0.85	0.084	0.845	0.866

Supplementary Table 15: Relationship between genome-wide significant SNPs genotype and patient overall survival[37]. Data from: 1,165 cases from the UK MRC Myeloma-IX trial (UK-MyIX); 877 MM cases from the UK MRC Myeloma-XI trial (UK-MyXI); 511 of the patients recruited to the German-GWAS (GER-GMMG); 703 MM cases in the UAMS Myeloma Institute for Research and Therapy GWAS (US-UAMS). *P* values calculated from Cox regression analysis.

SNP	Gene	Non silent mutations	Silent mutations	Noncoding mutation	*P* value	*q* value (FDR)
rs34229995	*DTNBP1*	0	1	0	0.184	1
rs34229995	*JARID2*	0	2	0	0.910	1
rs9372120	*ATG5*	0	1	0	1	1
rs7781265	*SMARCD3*	0	0	0	1	1
rs7781265	*CHPF2*	0	1	0	0.983	1
rs2811710	*CDKN2A*	0	0	0	1	1
rs2790457	*WAC*	0	1	0	0.760	1
rs7193541	*RFWD3*	0	0	0	1	1
rs6066835	*PREX1*	0	0	0	1	1

Supplementary Table 16: Mutation significance analysed with MutSigCV[38] on 463 MM exomes. The frequency of somatic mutation in genes annotated by GWAS signals was derived from tumor whole exome sequencing of Myeloma XI trial patients.

SNP	Locus	Gene 1	Gene 2	Pearson's Correlation (95% CI)	*P* value
rs9372120	6q21	*ATG5*	*PRDM1*	0.50 (0.29-0.66)	1.99×10^{-5}
rs2811710	9p21.3	*MTAP*	*CDKN2A*	0.54 (0.34-0.69)	3.13×10^{-6}
rs2811710	9p21.3	*CDKN2A*	*CDKN2B*	0.53 (0.33-0.68)	4.99×10^{-6}

Supplementary Table 17: A pair-wise correlation between gene expression (RNA-seq) within TADs. Analysis based on RNAseq transcript counts from 66 MM cell lines from the Keat's lab Data Repository[39] (Pearson product-moment correlation coefficient).

SNP	Reaction	Conditions		Sequence
rs34229995	Kaspar	Std42	Kaspar A1	GAAGGTGACCAAGTTCATGCTGCCCCATTGGATCCTTCCC
			Kaspar A2	GAAGGTCGGAGTCAACGGATTCTGCCCCATTGGATCCTTCCG
			Kaspar C	CACTTCACAATTGGAAAAATAAATGATCTT
	Sanger seq	Std	Seq F	ATTCCCAGTATGCCAGTCCT
			Seq R	AGGCACGTTATTGGAAGCTT
rs9372120	Kaspar	Std42	Kaspar A1	GAAGGTGACCAAGTTCATGCTCCTCCCTGGGTTACCAAATTCTC
			Kaspar A2	GAAGGTCGGAGTCAACGGATTACCTCCCTGGGTTACCAAATTCTA
			Kaspar C	ACCATTTTATATCAGACTCTCAAACATCAG
	Sanger seq	Std	Seq F	TGCACATTAACTCAGCCACA
			Seq R	GGAAACCGTATTTGAGTAGACCA
rs7781265	Kaspar	Std42	Kaspar A1	GAAGGTGACCAAGTTCATGCTCCCAATTCAGGGTCCCTGGAT
			Kaspar A2	GAAGGTCGGAGTCAACGGATTCCCAATTCAGGGTCCCTGGAC
			Kaspar C	CCCACCTTCCAGGGTGTGGCT
	Sanger seq	Std	Seq F	CCCATTAATGAGGTGGTCGC
			Seq R	CGATCCCCTTGTGTTTGCC
rs1948915	Kaspar	Std42plus5	Kaspar A1	GAAGGTGACCAAGTTCATGCTAGAGTTTTGTTTCCTTCCCACAGATAT
			Kaspar A2	GAAGGTCGGAGTCAACGGATTGAGTTTTGTTTCCTTCCCACAGATAC
			Kaspar C	ATGCCTTGTAATGTGCACCATTCTATGTA
	Sanger seq	Std	Seq F	CTCACGCAGATCACATGACC
			Seq R	GTGGGCGTCCTCACATTTTG
rs2811710	Kaspar	Std42	Kaspar A1	GAAGGTGACCAAGTTCATGCTTATAGCTACTTCAGAAGGCTCAGG
			Kaspar A2	GAAGGTCGGAGTCAACGGATTTATAGCTACTTCAGAAGGCTCAGA
			Kaspar C	CTTACCTTGAGAGGACTCTGTGCTA
	Sanger seq	Std	Seq F	AGTCTTGATTTCTGAAAGGGCTA
			Seq R	TTGTTACTTCTACCTTGGTGCA
rs2790457	Kaspar	Std42	Kaspar A1	GAAGGTGACCAAGTTCATGCTGTGGTGGTACCTGCCTTTAGTT
			Kaspar A2	GAAGGTCGGAGTCAACGGATTGTGGTGGTACCTGCCTTTAGTC
			Kaspar C	CCTTGACCTCCTCAGCTTAAGTGAT
	Sanger seq	Std	Seq F	TGCATTTCAGTGTGGGCTTT
			Seq R	AGTCATAACTCAACAAACCGTC
rs7193541	Kaspar	Std42	Kaspar A1	GAAGGTGACCAAGTTCATGCTGGACTGGCCAATGGTTCAG
			Kaspar A2	GAAGGTCGGAGTCAACGGATTGCTGGACTGGCCAATGGTTCAA
			Kaspar C	GACTGCTCGTGTTTCGCACGTCAT
rs6066835	Kaspar	Std42plus5	Kaspar A1	GAAGGTGACCAAGTTCATGCTGTGCCCAGTTCAATCACGCCC
			Kaspar A2	GAAGGTCGGAGTCAACGGATTGTGCCCAGTTCAATCACGCCT
			Kaspar C	TGCAGGGTCTCAGGCCTGAGAT
	Sanger seq	Std	Seq F	CTGGTGAGGAGGATTCTGGG
			Seq R	GGAACACTTGGACTCTGAGC
rs78311596	Kaspar	Std42	Kaspar A1	GAAGGTGACCAAGTTCATGCTGGAGATGGTCCTATGCTTTTAGGAT
			Kaspar A2	GAAGGTCGGAGTCAACGGATTGGAGATGGTCCTATGCTTTTAGGAA
			Kaspar C	TAAGGTCACTCACATGGTACGGGA
	Sanger seq	Std	Seq F	CGCTGTGCTCCAACATTCAT
			Seq R	CTGCAGCATTCCAGAGGCT
rs72665486	Kaspar	3stepStd42	Kaspar A1	GAAGGTGACCAAGTTCATGCTTTCCAGTTGAGCTTCGGATAAGAC
			Kaspar A2	GAAGGTCGGAGTCAACGGATTTTCCAGTTGAGCTTCGGATAAGAA
			Kaspar C	CACAAGACTGAGATCAAGGTAATCTTCAA
	Sanger seq	Std	Seq F	CACAGTTTGTTTTGGGTTAGGA
			Seq R	TTGAAGACCTAACGGCCCTA
rs1034447	Kaspar	Std42	Kaspar A1	GAAGGTGACCAAGTTCATGCTAGGAAAACTCCCATGATGCAGTG
			Kaspar A2	GAAGGTCGGAGTCAACGGATTACAGGAAAACTCCCATGATGCAGTA
			Kaspar C	GTCTGCACACATCTCCATGTGTATG
	Sanger seq	Std	Seq F	AGTGCCTAAATACATACCACCTT
			Seq R	TCAAGGACAAATGTTCCAGGT
rs17507636	Kaspar	Std42	Kaspar A1	GAAGGTGACCAAGTTCATGCTGTATAGGGTAGAATCTAATGCTCTGG
			Kaspar A2	GAAGGTCGGAGTCAACGGATTGTATAGGGTAGAATCTAATGCTCTGA
			Kaspar C	AGAAGTTCTTTCACTGTAGCCATCTGTAT
rs28571765	Kaspar	Std42	Kaspar A1	GAAGGTGACCAAGTTCATGCTCCTGTAATCCCAGCACTTTGAGG
			Kaspar A2	GAAGGTCGGAGTCAACGGATTACCTGTAATCCCAGCACTTTGAGA

			Kaspar C	TCTCAAACTCCTGGGCTCAAGCAAT
	Sanger seq	Std	Seq F	TCTTACTTCCATCACCCATGT
			Seq R	AGCTATACTATGAATCAAAGCA
rs76601148	Kaspar	Std42	Kaspar A1	GAAGGTGACCAAGTTCATGCTACAAAATGGGATATCCAATGATTACTACTA
			Kaspar A2	GAAGGTCGGAGTCAACGGATTCAAAATGGGATATCCAATGATTACTACTG
			Kaspar C	CCTGGATAAGATAATCACCGTGTTCATTT
	Sanger seq	Std	Seq F	TGTAGCGACTGGAAAATGGC
			Seq R	CCTGGATAAGATAATCACCGTGT
rs8058578	Kaspar	Std42	Kaspar A1	GAAGGTGACCAAGTTCATGCTTCAACTTCATATTGTTGGTGTTTTCTC
			Kaspar A2	GAAGGTCGGAGTCAACGGATTCTTCAACTTCATATTGTTGGTGTTTTCTT
			Kaspar C	GGGCGAAAAAGGAAGAGCCAAAGA
rs6071887	Kaspar	Std42plus5	Kaspar A1	GAAGGTGACCAAGTTCATGCTGAACAGAAGAAATAATAAATAGCTGATTGAGT
			Kaspar A2	GAAGGTCGGAGTCAACGGATTACAGAAGAAATAATAAATAGCTGATTGAGC
			Kaspar C	CGAGATTCTTGGAAGAAGGTTGATTACAT
	Sanger seq	Std	Seq F	CCAACCAGCAGAGTTTAGACG
			Seq R	ATGGAGTTCAGCTTGTGGGT

KASPAR conditions

Std42

- Hot Start: 94ºC for 15 minutes
- Stage 1: 20 cycles
  - 94ºC for 10 seconds
  - 57ºC for 5 seconds
  - 72ºC for 10 seconds
- Stage 2: 22 cycles
  - 94ºC for 10 seconds
  - 57ºC for 20 seconds
  - 72ºC for 40 seconds

Std42plus5

- Hot Start: 94ºC for 15 minutes
- Stage 1: 20 cycles
  - 94ºC for 10 seconds
  - 57ºC for 5 seconds
  - 72ºC for 10 seconds
- Stage 2: 22 cycles
  - 94ºC for 10 seconds
  - 57ºC for 20 seconds
  - 72ºC for 40 seconds
- Stage 3: 5 cycles
  - 94ºC for 10 seconds
  - 57ºC for 1 minute

3StepStd42

- Hot Start: 94ºC for 15 minutes
- Stage 1: 42 cycles
  - 94ºC for 10 seconds
  - 57ºC for 20 seconds
  - 72ºC for 40 seconds

Sequencing conditions

Std

- 95ºC for 5 minutes
- 25 cycles
  - 96ºC for 30 seconds
  - 50ºC for 15 seconds
  - 60ºC for 1 minute

Supplementary Table 18: Primers and probes and reaction conditions used for this study.

SUPPLEMENTARY REFERENCES

1. Rao, S.S. *et al.* A 3D map of the human genome at kilobase resolution reveals principles of chromatin looping. *Cell* **159**, 1665-80 (2014).
2. Swaminathan, B. *et al.* Variants in ELL2 influencing immunoglobulin levels associate with multiple myeloma. *Nat Commun* **6**, 7213 (2015).
3. Broderick, P. *et al.* Common variation at 3p22.1 and 7p15.3 influences multiple myeloma risk. *Nat Genet* **44**, 58-61 (2012).
4. Chubb, D. *et al.* Common variation at 3q26.2, 6p21.33, 17p11.2 and 22q13.1 influences multiple myeloma risk. *Nat Genet* **45**, 1221-5 (2013).
5. Swaminathan, B. *et al.* Variants in ELL2 influencing immunoglobulin levels associate with multiple myeloma. *Nat Commun* **6**, 7213 (2015).
6. Cozen, W., *et al.* "A meta-analysis of Hodgkin lymphoma reveals 19p13. 3 TCF3 as a novel susceptibility locus." *Nature Commun* **5**, 3856 (2014).
7. Zhang, Ben, *et al.* "Large-scale genetic study in East Asians identifies six new loci associated with colorectal cancer risk." *Nature genetics* **46,** 533-542 (2014).
8. Knipe, Duleeka W., *et al.* "Genetic Variation in Prostate-Specific Antigen–Detected Prostate Cancer and the Effect of Control Selection on Genetic Association Studies." *Cancer Epidemiology Biomarkers & Prevention* **23,** 1356-1365 (2014).
9. Lange, Ethan M., *et al.* "Genome-wide association scan for variants associated with early-onset prostate cancer." *PloS one* **9**, e93436 (2014).
10. Whiffin, Nicola, *et al.* "Identification of susceptibility loci for colorectal cancer in a genome-wide meta-analysis." *Human molecular genetics* **23**, 4729-4737 (2014).
11. Ahsan, Habibul, *et al.* "A Genome-wide association study of early-onset breast cancer identifies PFKM as a novel breast cancer gene and supports a common genetic spectrum for breast cancer at any age." *Cancer Epidemiology Biomarkers & Prevention* **23**, 658-669 (2014).
12. Gudmundsson, Julius, *et al.* "A common variant at 8q24. 21 is associated with renal cell cancer." *Nature communications* **4**, 2776 (2013).
13. Figueroa, Jonine D., *et al.* "Genome-wide association study identifies multiple loci associated with bladder cancer risk." *Human molecular genetics* **23**, 1387-1398 (2014).
14. Berndt, Sonja I., *et al.* "Genome-wide association study identifies multiple risk loci for chronic lymphocytic leukemia." *Nature genetics* **45**, 868-876 (2013).
15. Michailidou, Kyriaki, *et al.* "Large-scale genotyping identifies 41 new loci associated with breast cancer risk." *Nature genetics* **45**, 353-361 (2013).
16. Pharoah, Paul DP, *et al*. "GWAS meta-analysis and replication identifies three new susceptibility loci for ovarian cancer." *Nature genetics* **45**, 362-370 (2013).
17. Xu, Jianfeng, *et al.* "Genome-wide association study in Chinese men identifies two new prostate cancer risk loci at 9q31. 2 and 19q13. 4." *Nature genetics* **44**, 1231-1235 (2012).
18. Cheng, Iona, *et al.* "Evaluating genetic risk for prostate cancer among Japanese and Latinos." *Cancer Epidemiology Biomarkers & Prevention* **21**, 2048-2058 (2012).
19. Schumacher, Fredrick R., *et al.* "Genome-wide association study identifies new prostate cancer susceptibility loci." *Human molecular genetics* **20**, 3867-3875 (2011).
20. Sanson, Marc, *et al.* "Chromosome 7p11. 2 (EGFR) variation influences glioma risk." *Human molecular genetics* **20**, 2897-904 (2011).
21. Fletcher, Olivia, *et al.* "Novel breast cancer susceptibility locus at 9q31. 2: results of a genome-wide association study." *Journal of the National Cancer Institute* **103**, 425-35 (2011).
22. Takata, Ryo, *et al.* "Genome-wide association study identifies five new susceptibility loci for prostate cancer in the Japanese population." *Nature genetics* **42**, 751-754 (2010).
23. Gudmundsson, Julius, *et al.* "Genome-wide association and replication studies identify four variants associated with prostate cancer susceptibility." *Nature genetics* **41**, 1122-1126 (2009).

24. Shete, Sanjay, *et al.* "Genome-wide association study identifies five susceptibility loci for glioma." *Nature genetics* **41**, 899-904 (2009).
25. Tenesa, Albert, *et al*. "Genome-wide association scan identifies a colorectal cancer susceptibility locus on 11q23 and replicates risk loci at 8q24 and 18q21." *Nature genetics* **40**, 631-637 (2008).
26. Eeles, Rosalind A., *et al.* "Multiple newly identified loci associated with prostate cancer susceptibility." *Nature genetics* **40**, 316-321 (2008).
27. Enciso-Mora, Victor, *et al.* "Deciphering the 8q24. 21 association for glioma." *Human molecular genetics* **22**, 2293-302 (2013).
28. Stacey, Simon N., *et al.* "Germline sequence variants in TGM3 and RGS22 confer risk of basal cell carcinoma." *Human molecular genetics* **23**, 3045-53 (2014).
29. Xu, Heng, *et al.* "Inherited coding variants at the CDKN2A locus influence susceptibility to acute lymphoblastic leukaemia in children." *Nature communications* **6**, 7553 (2015).
30. Berndt, Sonja I., *et al*. "Genome-wide association study identifies multiple risk loci for chronic lymphocytic leukemia." *Nature genetics* **45**, 868-876 (2013).
31. Rajaraman, Preetha, *et al.* "Genome-wide association study of glioma and meta-analysis." *Human genetics* **131**, 1877-1888 (2012).
32. Barrett, Jennifer H., *et al.* "Genome-wide association study identifies three new melanoma susceptibility loci." *Nature genetics* **43**, 1108-1113 (2011).
33. Wrensch, Margaret, *et al.* "Variants in the CDKN2B and RTEL1 regions are associated with high-grade glioma susceptibility." *Nature genetics* **41**, 905-908 (2009).
34. Chiecchio, L. *et al*. *Leukemia* **20**, 1610–1617 (2006).
35. Neben, K. *et al*. *Haematologica* **95**, 1150–1157 (2010).
36. Kaiser, M. F., *et al*. *Leukemia* **27**, 1754-1757 (2013).
37. Johnson, David C., *et al*. "Genome-wide association study identifies variation at 6q25. 1 associated with survival in multiple myeloma." *Nature communications* **7**, 10290 (2016).
38. Lawrence, Michael S., *et al*. "Mutational heterogeneity in cancer and the search for new cancer-associated genes." *Nature* **499**, 214-218 (2013).
39. Keats, Jonathan J., *et al*. Keats Lab, http://www.keatslab.org/data-repository.
40. Westra, H.J. *et al*. Systematic identification of trans eQTLs as putative drivers of known disease associations. *Nat Genet* **45**, 1238-43 (2013).
41. Grundberg, E. *et al*. Mapping cis- and trans-regulatory effects across multiple tissues in twins. *Nat Genet* **44**, 1084-9 (2012).
